# Ultra-Cycling– Past, Present, Future: A Narrative Review

**DOI:** 10.1186/s40798-024-00715-7

**Published:** 2024-04-29

**Authors:** Lucas Tiemeier, Pantelis T. Nikolaidis, Daniela Chlíbková, Matthias Wilhelm, Mabliny Thuany, Katja Weiss, Beat Knechtle

**Affiliations:** 1 Centre for Rehabilitation & Sports Medicine, Bern University Hospital, Inselspital, University of Bern, Bern, Switzerland; 2 School of Health and Caring Sciences, University of West Attica, Athens, Greece; 3 Centre of Sports Activities, Brno University of Technology, 61669 Brno, Czech Republic; 4 Faculty of Sports, University of Porto, Porto, Portugal; 5 Institute of Primary Care, University of Zurich, Zurich, Switzerland; 6 Medbase St. Gallen Am Vadianplatz, Vadianstrasse 26, 9001 St. Gallen, Switzerland

**Keywords:** ultra-cycling, Race across America, Endurance, Performance

## Abstract

**Background:**

Ultra-endurance events are gaining popularity in multiple exercise disciplines, including cycling. With increasing numbers of ultra-cycling events, aspects influencing participation and performance are of interest to the cycling community.

**Main body:**

The aim of this narrative review was, therefore, to assess the types of races offered, the characteristics of the cyclists, the fluid and energy balance during the race, the body mass changes after the race, and the parameters that may enhance performance based on existing literature. A literature search was conducted in PubMed, Scopus, and Google Scholar using the search terms ‘ultracycling’, ‘ultra cycling’, ‘ultra-cycling’, ‘ultra-endurance biking’, ‘ultra-bikers’ and ‘prolonged cycling’. The search yielded 948 results, of which 111 were relevant for this review. The studies were classified according to their research focus and the results were summarized. The results demonstrated changes in physiological parameters, immunological and oxidative processes, as well as in fluid and energy balance. While the individual race with the most published studies was the Race Across America, most races were conducted in Europe, and a trend for an increase in European participants in international races was observed. Performance seems to be affected by characteristics such as age and sex but not by anthropometric parameters such as skin fold thickness. The optimum age for the top performance was around 40 years. Most participants in ultra-cycling events were male, but the number of female athletes has been increasing over the past years. Female athletes are understudied due to their later entry and less prominent participation in ultra-cycling races. A post-race energy deficit after ultra-cycling events was observed.

**Conclusion:**

Future studies need to investigate the causes for the observed optimum race age around 40 years of age as well as the optimum nutritional supply to close the observed energy gap under consideration of the individual race lengths and conditions. Another research gap to be filled by future studies is the development of strategies to tackle inflammatory processes during the race that may persist in the post-race period.

## Key Points

• The optimal ultra-cycling race age is around 40 years.
• Performance-related factors were the most investigated topic of ultra-cycling studies.
• There is a lack of information about strategies to tackle inflammatory processes during ultra-cycling events.

## Background

Ultra-endurance competitions are specific events extending over an exceptionally long distance and time. They are defined as distances longer than the classical marathon and longer than 6 h in duration [1]. Ultra-endurance events exist for different athletic disciplines, including running, cycling, and swimming [2]. Considering the duration of these events and the demanding preparation of athletes, ultra-endurance imposes extreme stress on the human body, which justifies the scientific interest in this topic.

Ultra-running events are defined as any race longer than a marathon of 42.195 km for distance-limited events and more than six hours for time-limited events [1] and ultra-triathlons as any race longer than the Ironman-distance triathlon (3.8 km swimming, 180 km cycling and 41.195 km running). In contrast, there is no comparatively precise definition of ultra-cycling. The Worldwide Ultracycling Association (WUCA) defines time-limited cycling races as those encompassing a challenge of at least six hours duration [1–3], while distance-limited events must stretch over at least 125 miles (200 km) and be completed in a single effort to be considered as an ultra-competition [3]. Thus, the definition of ultra-cycling is similar to that of ultra-running for time-limited events, whereas it differs for distance-limited events due to the faster nature of cycling compared to running.

Such ultra-endurance events have gained increasing popularity during the past 25 years due to the rise of master athletes [4] and the participation of female athletes [2, 5]. Nowadays, ultra-cycling competitions are offered around the globe in varying time and distance formats [6]. Moreover, due to geographical differences between the countries in which such races take place, the elevation and distances above sea level vary as well [6]. Possibly the most famous road ultra-cycling event at the moment is the Paris-Brest-Paris race [7]. The ‘Race Across America’ (RAAM) in the United States is currently the longest non-stop road ultra-cycling event [8]. It stretches over 4,800 km and has been taking place for over 40 years [9, 10]. The Death Ride in the United States is a 103 miles long road race with 14,000 feet of elevation (www.deathride.com) and ranks among the toughest races in the world. The Transcontinental Race self-supported ride across Europe with a distance of roughly 4,000 km, the Dragon Devil in Wales (320 km, almost 5,000 m of climbing), the Dirty Kanza XL (563 km, 4,500 m of elevation in Kansas), and the Mallorca 312 (312 km, 5,050 m elevation) are also considered as some of the most challenging ultra-distance cycling races on the planet (www.redbull.com/mea-en/worlds-toughest-endurance-cycling-events). The Great Divide (Tour Divide) is a mountain bike self-supported non-stop race with a 4,418 km distance from Canada to Mexico, the longest mountain bike race in the world. A non-stop and self-supported bicycle race held in Europe is the Transcontinental race, in which 4,000 km need to be covered (https://www.transcontinental.cc/about). Nowadays, the Silk Mountain Bike Race is considered the world’s hardest mountain bike race, an unsupported race in the Kyrgyzstan mountains with a length of 1,155 miles (www.silkmountainrace.com). The toughest winter races in the world are the Iditarod Trail Invitational race which takes part in Alaska where the participants travel along the historic Iditarod Trail on bicycle, foot, or skis (https://itialaska.com/iti-350) and the Arctic Circle Winter races in the fatbike category in the Finnish Lapland (http://www.arcticraces.com). The world’s toughest mountain bike races are La Ruta de los Conquistadores in Costa Rica with 29,000 feet of climbing over five mountain ranges and a 12,000-foot volcano (http://www.racelaruta.com) and the Trans Pyr in Spain involves eight days, 509 miles and over 66,601 feet of climbing (www.transpyr.com). Another mountain bike off-road ultra-cycling event is the Cape Epic mountain bike race, which entails a 16,650 m elevation gain and 624 km. In comparison, the road race RAAM includes the longest elevation gain with a 53,000 m climb along its course [8]. Among the world’s toughest mountain bike events, we can also list the Crocodile Trophy in Australia (eight days, 404 miles and 13,000 m of elevation), the Yak Attack in the Himalayas (10 stages, 310 miles and 15,000 m of elevation), the Iron Bike in Italy (seven stages, 435 miles, 22,000 m of overall climb) or the Tour d’Africa (90 stages, 7,450 miles, 71,564 m of overall climb).

Due to the increasing interest in participation in ultra-cycling races, numerous studies have been published in recent years addressing various aspects of such races, including the geographical differences between races, anthropometric characteristics of ultra-cycling participants, age- and sex-related differences between cyclists [2, 11] and methods to describe heart rate, exercise intensity and pacing [12–18]. In addition, research has been conducted on nutritional considerations [19–21], water and electrolyte disturbances during a race and rehydration [20, 22, 23], hematological and biochemical parameters [22, 24, 25], factors impacting on the performance (e.g., training status, fatigue and psychological parameters) [19, 26], and immunological and hormonal changes, in such a race [27, 28]..

## Main Text

### Aim

To date, there is no comprehensive literature review available on this topic summarizing the findings of such studies on ultra-cycling. Therefore, the aim of the present narrative review was to provide an overview of the available data and summarize the available information on ultra-cycling events and the participating cyclists. Topics of interest were geographic aspects of ultra-cycling races, characteristics of ultra-cyclists, age and sex differences, nutrition, fluid balance, body mass loss, and performance enhancement. The information provided can be useful for athletes and coaches to better delineate training strategies for ultra-cycling events.

## Methods

*Database and search strategy* A literature search was conducted in three databases: Scopus (https://www.scopus.com/home.uri), PubMed (https://pubmed.ncbi.nlm.nih.gov/), SPORTDiscus (https://www.ebsco.com/products/research-databases/sportdiscus), and Google Scholar (https://scholar.google.com/). The search terms used were ‘ultracycling’, ‘ultra cycling’, ‘ultra-cycling’, ‘ultra-endurance biking’, ‘ultra-bikers’ and ‘prolonged cycling’ and the search was conducted on December 31st, 2022.

Using this search strategy, 948 studies were identified, with 93 results in PubMed, 21 results in Scopus, 101 studies in SPORTDiscus, and 733 in Google Scholar. In Google Scholar, the search term ‘ultra-endurance cycling’ yielded no results, while ‘prolonged cycling’ yielded 15,900 results and was therefore deemed too unspecific and omitted from the Google Scholar search. Ten of the 21 studies identified in Scopus were duplicates of studies that had been already identified in PubMed. All 97 studies identified in PubMed and all 21 studies identified in Scopus were also identified using Google Scholar. All studies identified in SPORTDiscus had already been identified in PubMed and GoogleScholar. Two of the studies found in Scopus were excluded, because one was not available in either English or German, and the other was entirely off-topic.

### Study Eligibility

In addition to relevant studies listed in these databases, an online search was conducted for ultra-cycling events and road races around the world that meet the WUCA definition of ultra-cycling but may not have been identified in the scholarly databases. Studies published in English or German were considered. Studies focusing on a topic other than ultra-cycling were excluded, as were studies published in other languages. Studies reporting on triathlons, which typically entail a cycling distance of 40 km (standard triathlon) or 180 km (Ironman), were excluded, but ultra-triathlons covering a cycling distance of > 200 km in a single event were included, as these races qualify as an ultra-cycling event according to the WUCA definition.

Following the literature search, the relevant studies identified in the three databases were categorized based on their research focus according to the main topics of interest of the present narrative review: geographic aspects of ultra-cycling races, characteristics of ultra-cyclists, age- and sex-differences, nutrition, fluid balance, body mass loss, and performance enhancement (Table 1). Several studies focused on two or more of the areas of interest and are therefore listed twice or more frequently in each corresponding category of Table 1.

**Table 1:** Studies identified in PubMed and Scopus categorized by area of interest

Topic of interest	Number of studies	References
Geography/location	12	[9, 29–39]
Characteristics of ultra-cyclists	25	[2, 4, 9, 28, 33, 40–57]
Age differences	8	[14, 30, 58–63]
Sex differences	15	[23, 30, 38, 53, 59, 62, 64–72]
Nutrition	16	[73–90]
Fluid/mineral balance and rehydration	13	[23, 32, 91–101]
Body mass changes	14	[11, 22, 23, 27, 43, 44, 56, 91, 102–107]
Performance, physiology, hematological and biochemical parameters; thermoregulatory and humoral responses, injury, and performance enhancement, oxidative damage	41	[10, 13, 24, 26, 34–36, 52, 106, 108–139]
Endocrine responses	4	[35, 119, 120, 140, 141]
Cardiac function, exercise intensity and pacing, power output, VO_2_max	15	[12–18, 100, 142–148]

For each area of interest, the studies were accessed in full text, summarized, and the results compiled in the narrative review.

## Results

### Race Locations and Geographical Aspects

A total of 26 studies were identified in the search focusing on a particular race or a particular location. These specific races and the countries in which they took place are summarized in Table 2.

**Table 2:** Studies assessing races in different geographical regions

Country	Event	Distance and elevation	Reference
USA	RAAM	4,800 km, 25,000 m elevation	[9, 36, 90, 138, 139, 149]
France and Switzerland	Paris-Brest-Paris and RAAM qualifiers in Switzerland	600–720 km	[46]
France	Paris-Brest-Paris	1,230 km	[141]
Spain	Vuelta a España	3,431 km	[15, 16, 142, 145]
France	Tour de France	3,500 km	[16, 26, 106, 128, 142, 145, 146]
France	Paris-Roubaix	256,6 km	[150]
Italy	Giro d’Italia	3,448 km	[100, 131, 132, 142]
Italy	Milano-Sanremo	294 km, 2,000 m elevation	[151]*
Switzerland	Swiss Cycling Marathon	720 km, 4,700 m elevation	[30, 32]
Belgium	Tour of Flanders	237 km	[150, 152]
Belgium	E3 Saxo Bank Classic	203 km	[150]
Belgium	Liège-Bastogne-Liège	259 km	[150]
Netherlands	Amstel Gold	240 km	[153]*
Denmark - Italy	Copenhagen-Palermo	3,000 km	[63]
New Zealand	K4 cycle race	387 km	[89, 101]

*Note*: USA (the United States)

*Race found by online search, no scientific study pertaining to results of this race was identified

### The Ultra-Cyclist

Seventeen studies were identified that focused on the characteristics of ultra-cycling athletes (Table 3). In the beginning of ultra-cycling competitions, most participants were US nationals as the RAAM was the most competed in race [154]. With an increasing number of races taking place in Europe, the number of European participants in ultra-cycling races has also increased [154]. In a study investigating participation and performance trends in both an American qualifier (i.e., the Furnace Creek 508) and a European qualifier for the RAAM (i.e., the Swiss Cycling Marathon) and the RAAM itself, the characteristics of the participants and their performances were assessed [154]. Using a linear regression and variance analysis, it was shown that the races were very selective in terms of the number of racers participating and, ultimately, the resulting finishers. On average, ∼ 41% of participants did not finish either the RAAM or the Furnace Creek 508, whereas ∼ 26% did not finish the Swiss Cycling Marathon, with ∼ 26–40% of all starters not finishing the race [154]. Thus, the high rates of non-finishers highlighted the demanding nature of ultra-cycling races.

An overview of the participants in the identified primary studies is provided in Table 3, comparing cyclists’ characteristics such as age, sex, and training volume. Based on the existing literature incorporating the characteristics of ultra-cyclists, it appears that they are predominantly male and typically between 35 and 45 years old. Moreover, Swiss, German, and Scandinavian nationals dominate in European races, whereas North American cyclists are predominant in the RAAM [8, 31, 33, 40, 41, 154]. Compared to other individual ultra-disciplines like running or swimming, ultra-cyclists are slightly younger, have a slightly higher body mass index (BMI), and focus more on training volume than training intensity [44, 45].

**Table 3:** Characteristics of cyclists participating in ultra-cycling events

Event	Year	Participants	References
*Cycling events*			
Czech Championship 24-h mountain biking race	2013	Female winner, 46 years old, 15 years active, 12 finished ultra-races, 12 h training/week	[40]
Copenhagen-Palermo	2022	Comparison of two age groups (30 ± 5 years and 65 ± 6 years) in terms of metabolic and physiologic parameters	[63]
RAAM	2021	Male winner of RAAM, 75.2 h and 2,532 km completed as part of a team, average speed 35.9 km/h, average power 210 W, average heart rate 121 bpm	[139]
RAAM qualifier in Switzerland and Paris-Brest-Paris qualifier	2007–2009	Paris-Brest-Paris qualifier: mean age 45.2 years, BMI 24.2 kg/m^2^, higher training volume; RAAM qualifier: mean age 41.4 years, BMI 23.4 kg/m^2^, higher training intensity	[46]
RAAM	2018 (?)	4 female cyclists, 4 male cyclists, 7 male crew members, 1 female crew member, changes in physiological, psychological & perceptual parameters throughout the race in cyclists	[9]
Ötztal ultra-cycling race	1999	Single case study, male athlete, 36 years old, BMI 22 kg/m^2^, 12,000 km training in the year of the race	[49]
RAAM	2019 (?)	Single case study, female non-athlete, training from sedentary lifestyle to ultra-event	[28]
24 h time-limited, self-paced road race events	2022	Single case study of the biophysical characteristics of a male ultra-cyclist with two world records in ultra-cycling events	[57]
Cape Argus Cycling Tour and Cape Epic	2012	Differences between mountain bikers and road cyclists in ultra-races in terms of motivation to participate and behavior	[55]
*Ultra-triathlons*			
Double, Triple, and Deca Iron	1985 to 2009	Increase in overall number of finishers for Double (360 km cycling) and Triple (540 km cycling) races, Deca (1,800 km cycling) finishers stable, women relatively slower than men with increasing length	[42]
Triple Iron races vs. ultra-marathon races	2007–2009	Cyclists participating in triple triathlons (540 km cycling) are younger, have a higher training volume, and have higher body mass than runners	[44]
Ironman and Triple Ironman races	2007–2009	Ironman participants vs. Triple Ironman (540 km cycling) participants, 49 different nationalities, ultra-athletes with higher BMI, shorter stature, and larger training volume	[45]
Double Iron ultra-triathlon	1985–2010	European participants in ultra-triathlons (360 km cycling) have faster finishing times than North American athletes	[50]
Ultra-triathlon races	1985–2014	Influence of experience on performance in longer ultra-triathlons (360 km, 540 km, 900 km, or 1,800 km cycling)	[47]
Triple Iron ultra-triathlon races	1988–2011	Proportionally more of the female participants finished triple iron ultratriathlons (540 km cycling) than of the male participants, race time decreased for men, increased for women	[51]
Ultra-triathlon race	30 years’ time span	Two male athletes with 30 years’ experience in ultra-triathlon races (360–540 km cycling), > 60 years of age, performance decline highest for running discipline	[52]
Ultra-triathlon race	1985–2018	Fastest participants in ultra-triathlons (360 km, 540 km, 900 km, or 1,800 km cycling) are from Germany, France, and Switzerland; few North Americans competing in European races; less time for swimming and cycling, more time for running with increasing length of race	[53]

### The Aspect of Age in Ultra-Cycling Performance

Eight of the identified studies focused on the age of the cyclists as a potential factor of ultra-cycling performance (Table 1). Allen et al. compiled a systematic review and meta-analysis on the age of elite athletes at the peak of their performance [58]. They also included two studies on cycling events, one of which detailed the age of performers at the ultra-cycling events Furnace Creek and Swiss Cycling Marathon [154]. Allen et al. noted that the top performers in ultra-cycling events were on average 38 years old and thereby the oldest compared to the top performers in all other assessed sports, including marathon runners [58]. Knechtle et al. assessed whether the age of Ironman triathletes influences their performance and found that the younger athletes (18 to 40 years) were significantly slower in the cycling component of the event compared to the older age groups (> 50 years), while they were faster in the running and swimming events and had a shorter transition time [59].

A potential reason for this observation could be that the cycling performance declines relatively more slowly than the swimming performance [155]. Moreover, older athletes participating in ultra-races achieve a more even pacing compared to their younger competitors [59].

Lepers et al. analyzed the performance of participants in the Ironman Hawaii relative to their age at the time of competition [60]. Male and female finishers were categorized by their age and the performance of the top ten finishers in each group was evaluated over 15 years. During this period, a relative increase in athletes aged 40 years or older was observed, while a declining trend was noted for the age groups under 40 years. In addition, a trend was noted towards a continuously improving performance of the top finishers aged 45 years or older, and the sex differences in the top ten performers decreased with increasing age, in that the finishing times of the best ten male and female performers became more and more similar over the course of time.

Nikolaidis et al. assessed the age of peak performance of men and women participating in an ultra-distance duathlon, the Powerman Zofingen [61]. To do so, the authors divided the participants into age groups of five years and measured the effect of age on race time and on the type of race (short versus long version). The results showed that age affected the performance times in both the short and the long races, with younger athletes (25 to 29 years) completing the race significantly faster than the oldest athletes (70 to 74 years). Of note, the age of peak performance in the longer run (33 years) was higher than that in the shorter version (22 years) of the race, indicating that the older athletes performed well at a higher age in longer endurance competitions compared to the shorter distances. An explanation of this finding might be the accumulation of training and sport experience allowing the participation in longer races for older athletes.

Pozzi et al. investigated the age of peak performance of participants in a 24-hour ultra-cycling event in Switzerland over a 10-year period [156]. It was observed that the age of peak performance did not significantly change for male athletes over this period, but significantly decreased for females and for the overall participant pool. In turn, the achieved cycling distance was reduced with the increasing age of the athlete, and a significant effect of age on the performance was noted. The most successful participants were between 35 and 39 years old. Zingg et al. focused on the influence of age on the performance of participants in the Swiss Cycling Marathon and observed similar ages of the top performers, with an average of 35 years for men and 38 years for women [30].

The focus of a study by Wundersitz et al. was the physiological parameters during an ultra-cycling race relative to the age of the athletes [14]. The main outcome was the incidence of cardiac arrhythmias after the race. The results showed an overall increase in the incidence of cardiac arrhythmias, a decrease in aortic valve maximum velocity, and a slower mitral valve deceleration time. The incidence of cardiac arrhythmias was highest in the older age groups (45 years or older), rendering this group particularly susceptible to cardiovascular events during an ultra-cycling race.

Frandsen et al. conducted a 15-day intervention in which cyclists completed 3000 km over a time period of 15 days [63]. Before and after the intervention, VO_2max_, lipid parameters (resting plasma fatty acids, low density lipoprotein (LDL)-cholesterol, CD36, fatty acid binding protein/FABP), proteins involved in glucose metabolism (glucose transporter 4/GLUT4, SNAP23), and reactive oxygen species production were compared between younger (average age = 30 years) and older (average age = 65 years) participants. The results demonstrated that the adaptive response of younger cyclists was superior to that of older cyclists. Older cyclists exhibited impaired adaptation of their cardiovascular function due to the decreased adaptive response of metabolic parameters.

Summarizing the studies on the influence of age on performance in ultra-cycling events, it can be concluded that the highest performance is achieved in the mid- to late thirties, and that the finishing time decreases from the age of 40 years secondary to increased cardiac arrhythmia and decreased adaptation. In light of this observed decline in performance, metabolic and physiological problems occur more frequently in cyclists aged 40 or older, which likely contributes to a slower finishing time.

### Sex as a Factor in Performance

A total of 15 studies were identified that focused on sex differences between cyclists participating in ultra-cycling events.

Knechtle et al. analyzed the participants in Double, Triple, and Deca Iron ultra-triathlons over 25 years and found an overall decrease in the speed of female racers with increasing length of the race, which was not the case for male participants [42]. The number of female participants increased steadily from the first race held in 1985. The finish rate was higher for women than for men in the Double and Triple Iron ultra-triathlons, with 79.6% of men and 84.7% of women finishing the Double Iron ultra-triathlons and 71.7% of men and 77.0% of women finishing the Triple Iron ultra-triathlons. In turn, only 55.5% of female participants in the Deca Iron ultra-triathlons completed the race, while 82.9% of male participants finished these races.

For all three races, women took longer to complete the race. Comparing the best performance times of male and female participants, women required 21.2% more time than men to complete the cycling component of Double Iron ultra-triathlons, 23% more time than men to complete the cycling component of Triple Iron ultra-triathlons, and 19.4% more time to complete the cycling component of Deca Iron ultra-triathlons. These sex-related differences in performance times could be explained by anthropometric differences, e.g., the higher fat mass and lower skeletal muscle mass of women compared to men.

In contrast to the observation of these studies that the participation of female athletes in ultra-races increases, Rüst et al. observed that the number of male participants in Triple Iron ultra-triathlons significantly increased over a 13-year period, while that of female participants remained stable [51]. Moreover, in contrast to the study on the Norseman Xtreme race, the sex gap in terms of the finishing time increased over time from 10 to 42%. The authors speculated that this may be due to a diminished importance of the finishing time for women due to social factors.

Lepers and Stapley investigated the issue of sex differences in performance of participants in ultra-races and summarized the sex performance gap as 10% [4]. They identified physiological differences between men and women as potential factors influencing this performance gap, including the larger VO_2_max of men, lower body fat, greater muscle mass, and a greater hemoglobin concentration [4]. The authors noted that due to these physiological differences, biological factors are not responsible for the observed change in performance over time, i.e., the observed changes in the differences between women and men. They identified age as a potential factor to increase the sex gap, because less women participate in ultra-races in the higher age groups and, hence, fewer regular competitors are found among the female participators. Moreover, social factors may have prevented female participants from achieving their peak performance.

Scheer assessed participation trends in different ultra-races and identified an overall low participation of female athletes in ultra-cycling events, ranging between three and 11% [2]. It was noted, based on the existing research, that the proportion of females participating in ultra-cycling event is lower than in ultra-marathon running events [2]. In conclusion, sex-related differences in both the participation rates and performance in ultra-cycling events have been noted.

### Fluid and Electrolyte Balance and Rehydration

Twelve studies were identified in the literature search that focused on fluid and electrolyte balance and rehydration during an ultra-cycling event. Hyponatremia is defined as a sodium serum level below 135 mmol/l. Sodium serum levels below 120 mmol/l can cause neurological symptoms due to cerebral edema [157]. When sodium serum levels decrease below 110–115 mmol/l, respiratory failure due to cardiopulmonary decompensation may occur [158]. Different etiologies have been described for hyponatremia, including side effects of certain pharmaceuticals and systemic causes [159]. In terms of sporting events, hyponatremia correlates with sodium loss throughout a race, and this phenomenon is called exercise-associated hyponatremia (EAH) [160]. EAH occurs in intense training or competition with prolonged intense exertion for four to six hours or longer and is thus a concern for athletes participating in ultra-events [161].

Before EAH was conceptualized, athletes were generally recommended not to drink during an exercise, which led to hypernatremia [162]. Consequently, the American College of Sports Medicine unfortunately recommended drinking as much as possible during exercise to offset hyponatremia, thereby increasing the incidence of EAH in athletes due to fluid overload [162, 163]. In order to find a balance between hypo- and hypernatremia, fluid intake before, during, and after a race should be monitored to reach an optimum hydration status [164].

The literature search identified 11 studies focusing on fluid and electrolyte balance and rehydration during an ultra-cycling event. Moyen et al. conducted a study to determine the effect of the hydration status of participants in ultra-cycling events on their pain and mood [97]. They observed that throughout a race, fatigue, thirst, pain, thermal sensation, and exertion increased, while tension and vigor decreased. A comparison of dehydrated participants with those not suffering from dehydration showed that dehydration led to a stronger sensation of fatigue and pain at the beginning and in the middle of the race, whereas the hydration status did not affect any of the parameters after the race. Overall, dehydration was linked to more pain, fatigue, thirst, and thermal sensation compared to euhydration, while there was no significant effect of urine-specific gravity on the mood stage.

Black et al. assessed the fluid balance of ultracyclists participating in a 387-km race by analyzing blood and urine samples before and after the race [101]. Low blood sodium concentrations of 135 mmol/L were measured after the race, while dehydration only affected 2 of the 18 participants. The authors concluded that hyponatremia rather than dehydration is an issue that needs to be considered in the context of fluid balance of ultracyclists.

In a meta-analysis by Goulet and Hoffman, the effect of an *ad libitum* fluid intake during endurance events was compared to consumption of a pre-defined amount of liquids in terms of the participants’ performance [93]. Three studies on endurance cycling events were included in the analysis, and while none of these studies focused on ultra-races, an overall beneficial effect of liquid intake on pacing was noted, while an effect on performance was only observed for prescribed drinking and not for ad libitum intake [165–167]. It remains to be determined whether similar effects of the fluid intake can be observed for ultra-races. One study by Gauckler et al. investigated whether the drinking pattern during ultra-cycling races has effects on the incidence of edema [92]. A correlation between fluid intake pattern during the race and the occurrence of kidney-related symptoms, occurring in two-thirds of the participants, was determined. In this context, drinking as much as possible was positively correlated with the incidence of kidney-related problems and edema, while drinking according to the need based on the environmental conditions during a race showed a negative correlation with kidney-related symptoms and edema. These findings point towards an advantage of controlled fluid intake during an ultra-cycling event to prevent occurrence of limb swelling.

Chlíbková et al. assessed the hydration status of ultra-cyclists participating in mountain bike races [23]. While no changes in lower limb volume were observed after the race, the plasma urea level as an indicator of kidney function and water conservation negatively correlated with the ranking of the participants in the race, but this correlation was only significant for male participants.

Armstrong et al. determined the body water of participants in an ultra-cycling event of 164 km length [91]. The authors determined that changes in body mass and body water during the event were correlated but were not equivalent. However, body mass loss was concluded to be a suitable measurement to determine water loss during such an event and may serve as a basis for fluid replenishment.

In terms of hyponatremia during ultra-races, four studies were identified focusing on sodium levels during and after ultra-races. Knechtle et al. reviewed available data on the prevalence of hyponatremia and the associated clinical presentation and found evidence for a higher prevalence of hyponatremia in triathletes with increasing duration of the event (Ironman versus Triple Iron ultra-triathlon) [95, 98]. Moreover, a link between EAH and fluid overload was noted. In races where cycling was the only discipline, the prevalence of EAH appeared to be lower than in races combining multiple disciplines [95, 99].

### Nutrition, Energy Expenditure, and Performance

The literature search revealed 18 studies dealing with nutrition during an ultra-cycling event. These articles could broadly be divided into four focus areas: energy expenditure/energy balance during a race [73, 74, 78, 79, 83, 84, 90, 168], nutritional behaviour/food intake during a race [75, 77, 86, 89], the influence of nutrition on the performance [80, 82, 85, 141], and the breakdown of individual nutrients [76, 81].

### Energy Expenditure/Energy Balance

Barrero et al. conducted an analysis of the energy balance of ultra-endurance triathletes by assessing both the energy of the food and fluids consumed during the Extreme Man Salou-Costa Daurada triathlon in Spain and the expended energy according to heart rate, oxygen consumption, and body composition [73]. All participants were male and had been recreationally participating for at least three years in ultra-endurance events. The average energy intake amounted to 3,643 kcal, while the average energy expenditure was 11,009 kcal, demonstrating a significant energy deficit during the race. It was also observed that the athletes consumed significantly more macronutrients, solid foods, and fluids during the cycling stage of the race than during the running stage. Body mass significantly decreased, which was be primarily attributed to the loss of total body water. A similar energy deficit was observed by Bescós et al., conducting a case study on a single cyclist participating in a 24-hour-ultra-cycling event [74]. The athlete was male and predominantly consumed during the race solid foods rather than sports drinks, with a total energy intake of 5,571 kcal. The energy deficit amounted to 9,915 kcal, which was supposedly met by endogenous fuel stores. These values were similar to the energy expenditure determined for elite Ironman athletes of 9,626 kcal per race [83].

Enqvist et al. assessed the energy expenditure of athletes participating in a 800 km-adventure race with a cycling component by determining their heart rate and oxygen intake [78]. The average energy expenditure was 80,000 kcal, with a range of 64,000 to 114,000 kcal during the 5-day race. During an ultra-cycling race, an energy expenditure ranging from 10,557 kcal to 44,521 kcal was determined in five recreational athletes [79].

Hulton et al. determined the energy expenditure of cyclists participating in the RAAM [90]. The average energy expenditure amounted to 43,401 kcal and the daily energy expenditure to an average of 6,420 kcal. Based on the daily food and fluid intake, an energy deficit of 13,878 kcal was observed. A case report by Knechtle et al. also focused on the energy intake and energy expenditure of a cyclist participating in the RAAM [168]. The cyclist consumed 96,124 kcal in the course of the race, with 75.2% of these calories consumed as carbohydrates. The energy deficiency after the race amounted to 83,526 kcal., while it was not determined whether the 5 kg of body mass he lost during the race were due to protein (muscle) or fat metabolism.

A comparison of different types of ultra-endurance events revealed that cycling and triathlon races induce an approximately three times higher energy deficit compared with ultra-endurance running events [84]. In summary, ultra-cycling events lead to an energy deficit due to the discrepancy in energy intake and the extended energy expenditure. The optimum nutritional supply to close this energy gap has yet to be determined and must be adapted to the individual race lengths and conditions.

### Influence of Nutrition on Performance

In a study by Goedecke et al., the effect of supplying athletes during an experimental ultra-cycling race with a drink containing either carbohydrates alone or carbohydrates and medium-chain triglycerides on their performance [80]. The idea for this trial stemmed from the hypothesis that in the later stages of such races, when glycogen stores are depleted, lipids may serve as alternative energy sources. Eight male athletes participated in two trials in which one of the solutions was consumed. Performance was determined as the time to complete 75 kJ and 200 kJ trials. Both performance measures decreased significantly more when the solution contained carbohydrates and medium-chain triglycerides compared to carbohydrates alone. The reason for these performance differences could be the gastrointestinal symptoms and delayed gastric emptying experienced after medium-chain triglyceride consumption and the resultant heart rate increase early in the race. The overall fat content of the diet was a focus of a study by Rowlands and Hopkins, who compared the effect of a diet with a high fat content (70% of the total energy from fat) on the performance of competitive male ultra-cyclists with that of a diet with a high carbohydrate content (70% of the total energy from carbohydrates) [85]. The diets were consumed for two weeks prior to the experimental race. Performance in the ultra-cycling event was measured during a 100 km time trial. A trend was observed towards a better performance in the high-fat diet group compared to the high-carbohydrate diet group (4% improvement in time and 11.4% improvement in power output), but the difference lacked statistical significance. Nonetheless, the difference may have been statistically significant if the effect of both diets on performance had been compared in an ultra-endurance event.

The effect of carbohydrate supplementation in comparison with protein supplementation was assessed by Koopman et al., who focused on the protein turnover during an experimental ultra-endurance race with 2.5-hour bouts of cycling and intermittent one-hour running phases [82]. Two trials were conducted in which the same subjects consumed either a carbohydrate solution or a solution that contained both carbohydrates and protein hydrolysate. Protein turnover was measured by injecting amino acid isotope tracers. Supplementing the drink with protein hydrolysate resulted in an increased protein flux, protein synthesis rate, and whole-body protein oxidation compared to the drink containing carbohydrates only. Individual amino acid analysis revealed differences in the measurements of protein turnover, which in turn means that an effect of supplementation on protein metabolism depends on the composition of the protein hydrolysate. These results also demonstrate that protein supplementation induces protein metabolism more than carbohydrate supplementation and may therefore enhance performance during an ultra-endurance race.

Geesmann et al. evaluated the effect of energy balance of cyclists during an 1230-km ultracycling event on the release of hormones that are involved in metabolism [141]. They discovered that several metabolic hormones, including leptin and testosterone, were suppressed and remained suppressed for a 12-h-period following the end of the race. The suppression of insulin-like growth factor-1 correlated with the energy deficit during the race [141].

### Nutritional Behaviour/Food Intake

Bescós et al. studied the nutritional intake of male cyclists during a 24-hour-relay race in Barcelona [75]. While the athletes were encouraged to consume primarily carbohydrates before the race to fill their endogenous glycogen stores, they were allowed to consume any food of their choice during the race. They consumed mostly carbohydrate-rich foods and drinks during the event, with individual variations in the in-race intakes of protein and lipids. The intakes of sodium and caffeine increased towards the later stages of the race.

Chlíbková et al. determined the nutritional intake of ultra-cyclists before, during, and after a 24 h-ultra-cycling race [77]. Two-thirds of the athletes followed a diet rich in carbohydrates before the race, while the other third observed a protein- or fat-rich diet regimen. During the race, the athletes consumed 56 different foods and 16 different drinks. The most common foods were bananas, apples, oranges, raisins, pineapple, melon, chicken, bread, noodles, rice, biscuits, energy and muesli bars, cheese, chocolate, tomatoes, and dried fruits, while the most common drink was an isotonic sport drink followed by water. After the race, the food patterns were like the in-race pattern, with the most common foods being bread, noodles, rice, bananas, chicken, tomatoes, and cheese. The most common beverage was water. In a study assessing the nutritional habits of participants in the RAAM, similar foods were identified as in-race energy suppliers, primarily bananas, chicken, and bread [87]. The most common post-race foods were vegetables, cheese, and meat [87].

Wilson et al. performed an analysis of the types of saccharides contained in the carbohydrates consumed during an ultra-triathlon race [86]. Saccharide content was either measured by liquid chromatography or based on the values reported in the US Department of Agriculture Database. The proportions of glucose, fructose, and sucrose were calculated from 80 different consumed foods, and amounted to a median of 64%, 50%, and 10% of total carbohydrates, respectively.

Black et al. conducted a study on the energy intake of cyclists participating in a 384-km ultraendurance race [89]. The cyclists reported their food and fluid intake after the race and the nutrient composition of the consumed food and drinks was assessed. The results demonstrated an inverse correlation between the completion time and energy intake, as well as a correlation between carbohydrate and fat intake and completion time. The authors concluded that the energy requirement during such an ultraendurance event should be met with a high energy intake during the race in order to achieve the optimal performance.

### Individual Nutrients

Two studies assessed changes in the metabolism of individual nutrients during ultra-endurance events. Borgenvik et al. determined the amino acid metabolism of male participants in an adventure race and during post-race recovery [76]. They discovered that the plasma concentration of glutamine, glycine, lysine, serine, threonine, and valine was diminished by the race, while the concentration of tyrosine, phenylalanine and glutamate increased. In the muscle, only lysine significantly decreased. The post-exercise levels of branched-chain amino acids correlated with the glycogen content of the muscle, indicating a potential protein breakdown during the race due to depleted glycogen stores. No significant decreases in the amino acid levels were observed during the recovery phase.

Konopka et al. analyzed protein synthesis in the skeletal muscle during the ColoradoTrail Race, an ultra-endurance mountain bike race, and compared the data to a control situation without normal activity [81]. The authors discovered that protein synthesis in the skeletal muscle was increased during the race, and indices of cellular energetic stress increased.

Summarizing the results of studies focusing on nutrition during ultra-cycling events, it becomes obvious that there is a lack of studies on the nutritional behaviour and energy expenditure of female athletes, as all the studies were conducted with male athletes. Moreover, the question remains whether nutrients other than carbohydrates could enhance performance, particularly during the later stages of the race. At present, it remains unclear whether in-race triglyceride or protein supplements can contribute to a faster race time.

### Changes in Body Mass

The literature search retrieved 14 studies focusing on changes in body mass during an ultra-cycling race.

Bischof et al. determined anthropometric parameters of cyclists participating in the Swiss Cycling Marathon [102]. Anthropometric measurements included skinfold thickness, limb circumferences, and body mass. The relative proportion of body fat and muscle mass was calculated from these measurements. The anthropometric measurements were repeated at multiple checkpoints throughout the race. The results demonstrated a mean decrease in body mass by 1.5 kg and a decrease in fat mass of 1.5 kg. Skeletal muscle mass and estimated body water did not significantly change during the race. Decreases in skinfold thickness were noted for all measured limbs, with the abdominal skinfold thickness negatively correlating with the cycling speed. A potential explanation for these changes includes the degradation of subcutaneous adipose tissue in the body parts that are used most during an ultra-cycling event, such as the thigh and the pectoral muscles.

Chlíbková et al. aimed at determining the changes in body composition of ultra-cyclists during two 24-hour races [23]. Anthropometric measurements included skinfold measurements, limb circumferences, fat and muscle mass, body mass, as well as body water. Moreover, the changes in foot volume were assessed by plethysmography to determine the presence of edema. Changes in body mass correlated negatively with the distance covered during the race and positively with their overall ranking. The ranking also correlated positively with changes in fat mass and negatively with fluid intake. The absolute changes in body mass and fat and muscle mass were calculated separately for male and female participants. Male participants lost an average of 2.0 kg of body mass, 1.4 kg of fat mass, and 1.4% of body fat. Skeletal muscle mass did not decrease significantly. Female participants lost 0.9 kg of body mass during the race, 1.2 kg of fat mass, and 2.7% of body fat, while no significant changes in skeletal muscle mass were observed. No significant changes in the foot volume were noted in either men or women. The higher changes in the body mass of men compared to women could be explained by the fact that men had a higher body mass to start with, that they completed the race faster, and that men utilize intramyocellular lipids more than women [169, 170].

Knechtle et al. conducted several studies and on body mass and body composition changes during ultra-endurance events [11, 104, 105]. A case study of a participant of a Deca Iron Triathlon revealed a decrease in body mass of 1 kg, a decrease in fat mass by 0.8 kg, and a decrease in muscle mass by 0.9 kg [104]. In contrast, an assessment of eight athletes participating in the Deca Iron Triathlon showed no significant loss in body mass [103]. Losses of skeletal muscle mass (1.1 kg) and fat mass (0.9 kg) were like the losses observed in the case study [104].

A correlation between the intensity of the race and the body composition changes was noted in a study assessing body mass and composition changes of participants in the Triple Iron ultra-triathlon in Germany 2006 [105]. Body mass, limb circumferences, skinfold thicknesses, fat and muscle mass, and body fat percentage were measured before and after the race. A significant decrease in body mass of 3.9% was noted, as well as a decrease in limb circumferences for all body parts except the thigh and the chest. There was no significant change in skeletal muscle mass, yet both the BMI and the relative proportion of body fat decreased significantly. Along with these observations, no decrease in skeletal muscle mass was observed in participants in the Swiss Cycling Marathon [11]. Anthropometric measurements were conducted before and after the race. Body mass decreased by 1.7 kg and fat mass by 1.4 kg, while total body water and skeletal muscle mass showed no significant changes. The changes in body mass and fat mass correlated significantly, as did the changes in body mass and total body water changes. In a review assessing the changes in anthropometric measurements during ultra-endurance races, decreases in all assessed skinfolds (pectoral, mid-axilla, triceps, subscapular, abdominal, suprailiacal, thigh, and calf) were noted for ultra-endurance cyclists [43].

Valenti et al. assessed whether participation in a 4,400 km ultra-cycling race (NorthCape4000) impacts on body composition and metabolic parameters involved in myogenesis [27]. They conducted bioelectrical impedance analyses and dual energy x-ray absorptiometry to measure body composition before and after the race and determined the level of myogenic progenitor cells indirectly by the level of the transcription factors required for this process. Fat mass, visceral adipose tissue, and truncal fat significantly decreased after the race, while lean mass significantly increased [27]. The expression of transcription factors involved in myogenesis was elevated after the race.

### Performance, Injury, and Performance Enhancement

Forty-one studies were filtered from the search results that focused on factors influencing performance in an ultra-cycling race. Five themes pertaining to their influence on performance were identified: physiological, demographic and anthropometric parameters, pacing strategies, injury and inflammation, and psychological aspects of sleep. These themes and the respective studies are presented in Table 4. In terms of the physiological function, the findings were inconsistent in terms of the heart rate changes during the race, with an initial decrease observed in one study [110] and an initial increase observed in another study [121]. Male sex correlated positively with a better finish time [115], while anthropometric measurements were not associated with performance [114, 127].

In-race changes in inflammatory markers such as cytokines were observed in several studies [35, 112, 118–120, 124]. Oxidative changes induced by exercise could be modified by the dietary consumption of antioxidants [112], and inflammation was counter-balanced by taking anti-inflammatory medication [111]. Taking naps during an extended race resulted in a diminished performance in terms of the finish time [117]. Emotions experienced during the course of an extended race could exert both a stimulating and impairing effect on the performance, depending on the emotional state and the perceived emotional optimum state of the individual cyclist [36].

**Table 4:** Factors influencing performance in ultra-cycling or mixed ultra-endurance events

Race characteristics	Performance indicator	Outcome	Reference
Physiological function			
8.5 h, 105 km wilderness race	Respiratory function pre- and post-race, race completion in one athlete	Decrease in oxygen saturation, forced expiratory volume, forced vital capacity, potential airflow obstruction, no obvious respiratory symptoms, race completed	[123]
Ultra-cycling event	heart rate, cycling power, speed, distance	861.6 km completed, average power = 210 W, average heart rate = 121 bpm, greater power during daytime	[139]
RAAM	VO_2max_, lactate, power	pyramidal training intensity distribution-based training only marginally increases performance	[138]
Ultra-triathlon	Performance, heart rate, oxygen consumption relative to race intensity	Lower heart rate during cycling leg than during swimming leg, race time associated with difference in oxygen consumption and heart rate between running and swimming, but not cycling legs	[108]
24 h-ultra-cycling relay race	Physiological performance-related parameters and influence of team size	Relative power output positively correlated with covered distance and velocity; no higher average intensity observed relative to number of participants	[13]
Transcontinental Race 5	Heart rate and performance of a single athlete	Decrease in heart rate to mid-race, increase towards the end, effect of temperature and sleep quality on performance	[110]
Experimental 24-h race with running, cycling, and kayaking	Physiological parameters during mixed endurance race	Initial increase (up to 6 h) in heart rate during cycling leg, then decreased, oxygen consumption increased up to 6 h and remained high until the end	[121]
***Demographic and anthropometric parameters***			
Ironman Triathlon, Ultra-triathlon	Predictors of performance, finish time	Influence of sex, age, experience, and performance in cycling and running legs on finish time	[115]
Double and Triple Iron ultra-triathlons	Performance changes in two athletes over three decades	0.19% and 1.12% performance decline in cycling per year, largest declines in running discipline	[52]
Triple Iron Ultra-triathlon	Influence of anthropometric variables and split times for each discipline on performance	No significant correlation between anthropometric parameters and finish time, performance in running and cycling, but not swimming, influences finish time	[114]
RAAM	Differences between finishers and non-finishers	Anthropometric differences observed, finishers have lower BMI, lower percentage of body fat, and lower thigh and upper arm circumference than non-finishers, but anthropometric measurements not related to finish time	[127]
***Pacing***			
RAAM	Influence of pacing on performance over five year-period	Decrease in performance with increasing age and altitude changes, top finishers started faster, had higher peak speeds, and maintained speed longer	[10]
24-h ultra-cycling event	Influence of pacing on performance	Wind speed, temperature influence performance, pacing with beneficial impact on top performance	[116]
Swissultra	Pacing strategies in different multi-stage ultra-triathlon events	Race distance affects running, but not cycling performance in multi-stage events, pacing strategies affected by race distance	[12]
***Injury and inflammation***			
Ultra-cycling races	Shermer’s Neck in ultra-cyclists	Incident Shermer’s neck in ultra-cycling races after 800 km, prevention by stretching and chin support	[109]
Ultraman Florida Triathlon	Inflammatory markers pre- and post-race, finish time	Increase in C-reactive protein, no change in interleukins 6 and 10, positive correlation between interleukin 10 levels and finish time	[124]
24-h-mountain bike race	Intake of anti-inflammatory medication before, during, and after the race	10% of participants used anti-inflammatory medication during the race, mostly ibuprofen, users were significantly older than non-users	[111]
Experimental cycling test with triathletes and marathon runners	Effect of anti-oxidant consumption on performance	Changes in endogenous oxidant and anti-oxidant processes during the test, dietary anti-oxidants effective against exercise-induced oxidative stress	[112]
Hotter’N Hell Hundred	Inflammatory markers pre- and post-race, influence of pacing strategies on these markers	Increase in pro- and anti-inflammatory cytokines, shorter self-pacing associated with higher anti-inflammatory cytokine concentration	[119]
Hotter’N Hell Hundred	Immunological and hormonal changes during a race in hot climate and influence on performance	Testosterone decreased, growth hormone, cortisol, interleukin 6 increased, cortisol and growth hormone levels higher in fastest compared to slowest participant	[120]
Southern Traverse Adventure Race	Influence of heart rate, immunological parameters on performance	Heart rates declined up to mid-race and then remained stable, leukocyte plasma concentrations and plasma volume increased	[118]
***Psychological aspects and sleep***			
Ultra-cycling race	Effect of intermittent naps on performance	In-race naps decrease performance, low body fat and duration per training unit increase performance	[117]
5 h timed race with ultra-cyclists	Impact of mental workload on performance	Mental workload, quantified by EEG theta power, heart rate variability, and psychomotor vigilance, does not affect performance in a staged ultra-cycling race	[137]
10 km simulated Road to Camarón race	Sleep patterns of an ultra-cyclists before, during, and after the race	Sleep duration reduced during the race, slow-wave sleep increased	[136]
RAAM	Impact on in-race emotions (Brunel Mood Scale) and sleep on performance	Individual differences in emotional stage, fatigue and negative or positive emotions may enhance or diminish performance depending on personal optimal emotional state, fluctuation of emotions throughout the race	[36]

## Discussion

The present review aimed at assessing the current literature about ultra-cycling races. The results showed that the popularity of these events has been consistently increasing in recent years, with races being mainly offered in North America, Latin America, and Europe. While men dominate all races in terms of their proportion of the participants, the number of female cyclists is increasing.

Due to the later entry of women in such events, the available studies assess predominantly male athletes and, hence, there is a lack of knowledge on female athletes pertaining to their anthropometric measurements, their immunological and physiological parameters during a race, and the factors influencing their performance. The studies revealed changes in inflammatory and oxidative processes during an ultra-cycling race, which could impact performance. In particular, older athletes try to compensate for such changes by taking anti-inflammatory medication during the race, yet it remains unclear to what extent such an intake can prevent inflammatory processes during and after the race. The optimum fluid and nutrient intake before, during, and after the race is associated with the total body water and, hence, the hydration status and energy balance of the athlete. The best strategy to prevent hyponatremia of ultra-cyclists remains to be determined. EAH appears to be less of an issue for ultra-cyclists than for other ultra-athletes, potentially due to a more efficient control of fluid intake during a race, but the reason for these differences has yet to be clarified. Another open question regarding hyponatremia in ultra-cycling is the apparent lack of severe neurological consequences in hyponatremic ultra-cyclists despite critically low sodium levels [171].

It appears that many ultra-cyclists already focus on maximizing their glycogen stores before the race and consuming carbohydrate- and protein-rich foods before and after it, yet an energy deficit is frequently observed. Therefore, future studies should focus on the best nutritional preparation for an ultra-cycling event. The observed energy deficit may be due to the fact that nutrient oxidation exceeds nutrient transport in the gut, and hence, enhancing transport capacity of intestinal transporters may be an approach to diminish this deficit. It seems that carbohydrates are the most efficient nutrient for energy supply, with no benefit of pre- or in-race triglyceride supplementation being observed.

The available data on body composition indicate that an ultra-cycling event leads to decreases in body mass and fat mass, while skeletal muscle mass does not appear to significantly change. Nonetheless, study results differ, with decreases in muscle mass observed in some studies [103, 104] and no changes in total body water observed in studies with a loss in body mass [11]. Body mass losses occur as a consequence of ultra-cycling events, and these losses are higher in men than in women. These changes are putatively mostly due to the in-race degradation of lipids stored intramyocellularly or in the subcutaneous tissue. Potential explanations could be that men and women exhibit distinct changes in body mass, and that the anthropometry of athletes differs between individual sports and combined races such as triathletes. Therefore, depending on the participants that are studied, different outcomes in terms of body mass changes and composition may be expected. It is worthwhile investigating in future studies if and why skeletal muscle mass appears to be unaltered by ultra-endurance exercise, and whether the loss in body mass is solely attributable to the loss in total body water.

It must be acknowledged that there is no clear trend for consistently observed differences between the sexes, as studies found conflicting results. This may be due to a lack of comparability between these studies or the races described therein. Several open questions regarding the role of sex in race performance remain, including the observed differences in the peak performance age and the reasons for a comparatively lower performance improvement of participating women.

Topics that warrant further investigation are the causes for the optimum race age around 40 years of age. Judging from other sports, one would expect that younger cyclists would have a greater physical fitness and, hence, would have an advantage over the 40-year-olds. Nonetheless, experience might be a relevant factor for an optimum performance and could outweigh potential advantages of a younger age. In addition, the mitochondrial volume may reach an optimum in middle-aged athletes as demonstrated in animal studies [172]. This in turn could lead to diminished mitochondrial function and oxidative capacity to process nutrients, and be linked to a decrease in muscle mass and sarcopenia [173]. Moreover, it has been suggested that the accumulated experience leads to a reset of central fatigue [174].

Research gaps pertaining to influencing factors on performance in ultra-cycling races are interventions to tackle inflammatory processes during the race that may persist in the post-race period. Moreover, the currently available studies on physiological function are difficult to compare, because different race durations and intensities were assessed, mostly with only a few participants. In order to fully understand heart rate and oxygen consumption changes during ultra-cycling races, the intensity and the number of participants must be considered.

## Conclusions

In conclusion, literature on ultra-cycling races is comparatively scarce, yet certain aspects, such as the types of athletes and fluid balance/hyponatremia have been investigated. Future studies should primarily focus on the energy balance, the occurrence and consequences of inflammatory and oxidative processes, and the female athletes, as these aspects were identified as research gaps in this review. Topics that warrant further investigation include the causes for an optimum age of peak performance around 40 years and the optimum nutritional supply to close the observed energy gap under consideration of the specific race lengths and climate conditions. Another research gap to be closed by future studies is the development of strategies to tackle inflammatory processes during the race that may persist in the post-race period.

## Data Availability

not applicable.

## Abbreviations

BMI: body mass index
EAH: exercise-associated hyponatremia
FABP: fatty acid binding protein
GLUT4: glucose transporter 4
LDL: low density lipoprotein
RAAM: Race Across America
WUCA: Worldwide Ultracycling Association

## References

[1] Zaryski C, Smith DJ. Training principles and issues for ultra-endurance athletes. Curr Sports Med Rep. 2005;4(3):165–70. 10.1097/01.csmr.0000306201.49315.73. Epub 2005/05/24. 15907270 10.1097/01.csmr.0000306201.49315.73

[2] Scheer V. Participation Trends of Ultra Endurance Events. Sports medicine and arthroscopy review. 2019;27(1):3–7. Epub 2019/01/03. 10.1097/jsa.0000000000000198. PubMed PMID: 30601393. 10.1097/JSA.0000000000000198 30601393

[3] WUCA. What is ultracycling? 2022 [15.07.2022]. https://www.ultracycling.com/what-ultracycling.

[4] Lepers R, Stapley PJ. Master athletes are extending the limits of human endurance. Front Physiol. 2016;7:613. 10.3389/fphys.2016.00613. Epub 2016/12/27. 28018241 10.3389/fphys.2016.00613 PMC5149541

[5] da Fonseca-Engelhardt K, Knechtle B, Rüst CA, Knechtle P, Lepers R, Rosemann T. Participation and performance trends in ultra-endurance running races under extreme conditions - ‘Spartathlon’ versus ‘Badwater’. Extrem Physiol Med. 2013;2(1):15. 10.1186/2046-7648-2-15. PubMed PMID: 23848985. 23848985 10.1186/2046-7648-2-15 PMC3710197

[6] WUCA. World Ultracycling Association Race. Calendar 2022 [10. October 2022]. https://www.ultracycling.com/calendar.

[7] Apidura. Paris-Brest-Paris: Everything You Need to Know 2022 [11.07.2022]. https://www.apidura.com/journal/paris-brest-paris-everything-you-need-to-know/.

[8] RAAM. Race across America 2022 [11.07.2022]. https://www.raamrace.org/about.

[9] Guex K, Serain E, Gremion G, Besson C, Faiss R, Majo J, et al. Participating in the race across AMerica in a team of eight cyclists: do not neglect Crew Preparation. Open Access J Sports Med. 2019;10:161–9. 10.2147/oajsm.S219124. Epub 2019/12/07. 31807092 10.2147/OAJSM.S219124 PMC6839592

[10] Heidenfelder A, Rosemann T, Rüst CA, Knechtle B. Pacing strategies of Ultracyclists in the race across AMerica. Int J Sports Physiol Perform. 2016;11(3):319–27. 10.1123/ijspp.2015-0051. Epub 2015/07/29. 26215121 10.1123/ijspp.2015-0051

[11] Knechtle B, Wirth A, Knechtle P, Rosemann T. An ultra-cycling race leads to no decrease in skeletal muscle mass. Int J Sports Med. 2009;30(3):163–7. 10.1055/s-0028-1104585. Epub 2009/02/10. 19199212 10.1055/s-0028-1104585

[12] Weiss K, Sousa CV, Thuany M, Cuk I, Nikolaidis PT, Knechtle B. Differences in pacing during cycling and running in ultra-triathlons - the example of ‘Swissultra’. Eur Rev Med Pharmacol Sci. 2022;26(14):4959–68. Epub 2022/08/03. doi: 10.26355/eurrev_202207_29281. PubMed PMID: 35916791. 35916791 10.26355/eurrev_202207_29281

[13] Bescós R, Rodríguez FA, Iglesias X, Knechtle B, Benítez A, Marina M, et al. Physiological demands of cyclists during an ultra-endurance relay race: a field study report. Chin J Physiol. 2011;54(5):339–46. Epub 2011/12/06. PubMed PMID: 22135913. 22135913

[14] Wundersitz D, Williamson J, Nadurata V, Nolan K, Lavie C, Kingsley M. The impact of a 21-day ultra-endurance ride on the heart in young, adult and older adult recreational cyclists. Int J Cardiol. 2019;286:137–42. 10.1016/j.ijcard.2019.03.016. Epub 2019/03/25. PubMed PMID: 30904280. 30904280 10.1016/j.ijcard.2019.03.016

[15] Earnest CP, Jurca R, Church TS, Chicharro JL, Hoyos J, Lucia A. Relation between physical exertion and heart rate variability characteristics in professional cyclists during the Tour of Spain. Br J Sports Med. 2004;38(5):568–75. 10.1136/bjsm.2003.005140. Epub 2004/09/25. 15388541 10.1136/bjsm.2003.005140 PMC1724921

[16] Lucia A, Hoyos J, Santalla A, Earnest C, Chicharro JL. Tour De France versus Vuelta a España: which is harder? Med Sci Sports Exerc. 2003;35(5):872–8. 10.1249/01.Mss.0000064999.82036.B4. Epub 2003/05/17. 12750600 10.1249/01.MSS.0000064999.82036.B4

[17] Padilla S, Mujika I, Santisteban J, Impellizzeri FM, Goiriena JJ. Exercise intensity and load during uphill cycling in professional 3-week races. Eur J Appl Physiol. 2008;102(4):431–8. 10.1007/s00421-007-0602-9. PubMed PMID: 17978835. 17978835 10.1007/s00421-007-0602-9

[18] Neumayr G, Pfister R, Mitterbauer G, Gaenzer H, Sturm W, Hoertnagl H. Heart rate response to ultraendurance cycling. Br J Sports Med. 2003;37(1):89–90. 10.1136/bjsm.37.1.89. Epub 2003/01/28. 12547753 10.1136/bjsm.37.1.89 PMC1724597

[19] Linderman J, Demchak T, Dallas J, Buckworth J. Ultra-endurance cycling: a field study of human performance during a12-hour moutain bike race. J Exerc Physiol Online. 2003;6:14–23.

[20] Geesmann B, Mester J, Koehler K. Energy balance, macronutrient intake, and hydration status during a 1,230 km ultra-endurance bike marathon. Int J Sport Nutr Exerc Metab. 2014;24(5):497–506. 10.1123/ijsnem.2013-0169. Epub 2014/03/29. 24668685 10.1123/ijsnem.2013-0169

[21] May GC, Doherty AR, Smeaton AF, Warrington GD. Correlating multimodal physical sensor information with biological analysis in ultra endurance cycling. Sens (Basel). 2010;10(8):7216–35. 10.3390/s100807216. Epub 2010/01/01. 10.3390/s100807216 PMC3231179 22163600

[22] Stuempfle KJ, Lehmann DR, Case HS, Hughes SL, Evans D. Change in serum sodium concentration during a cold weather ultradistance race. Clin J Sport Med. 2003;13(3):171–5. 10.1097/00042752-200305000-00008. Epub 2003/06/07. 12792212 10.1097/00042752-200305000-00008

[23] Chlíbková D, Knechtle B, Rosemann TJ, Žákovská A, Tomášková I, Shortall M, et al. Changes in foot volume, body composition, and hydration status in male and female 24-hour ultra-mountain bikers. J Int Soc Sports Nutr. 2014;11:12. 24661412 10.1186/1550-2783-11-12 PMC3994394

[24] Valenzuela PL, Foster C, Lucía A, de la Villa P. Performance and physiological analysis of 500 km non-stop cycling: a case study. Res Sports Med. 2018;26(2):222–9. PubMed PMID: 29359592. 29359592 10.1080/15438627.2018.1431538

[25] Wirnitzer KC, Faulhaber M. Hemoglobin and hematocrit during an 8 day mountainbike race: a field study. J Sports Sci Med. 2007;6(2):265–6. Epub 2007/01/01. PubMed PMID: 24137084; PubMed Central PMCID: PMCPMC3786250. 24137084 PMC3786250

[26] Lucia A, Hoyos J, Chicharro JL. Physiology of professional road cycling. Sports Med. 2001;31(5):325–37. 10.2165/00007256-200131050-00004. Epub 2001/05/12. 11347684 10.2165/00007256-200131050-00004

[27] Valenti MT, Braggio M, Minoia A, Dorelli G, Bertacco J, Bertoldo F, et al. Effects of a 4400 km ultra-cycling non-competitive race and related training on body composition and circulating progenitors differentiation. J Transl Med. 2022;20(1):397. 10.1186/s12967-022-03591-5. Epub 20220904. 36058924 10.1186/s12967-022-03591-5 PMC9441096

[28] Guex K, Wicht S, Besson C, Degache F, Gojanovic B, Gremion G. From sedentary and physical inactive behaviours to an Ultra Cycling race: a mixed-method case report. Int J Environ Res Public Health. 2020;17(2). 10.3390/ijerph17020502. PubMed PMID: 31941104; PubMed Central PMCID: PMCPMC7014053. Epub 2020/01/17. 10.3390/ijerph17020502 PMC7014053 31941104

[29] Knechtle B, Rüst CA, Rosemann TJ, Martin N. 33 ironman triathlons in 33 days–a case study. Springerplus. 2014;3. 10.1186/2193-1801-3-269 PMC4047275 24926424

[30] Zingg M, Knechtle B, Rüst CA, Rosemann T, Lepers R. Age and gender difference in non-drafting ultra-endurance cycling performance - the ‘Swiss Cycling Marathon’. Extrem Physiol Med. 2013;2(1):18. 10.1186/2046-7648-2-18. Epub 2013/07/16. 23849106 10.1186/2046-7648-2-18 PMC3710092

[31] Rüst CA, Knechtle B, Rosemann T, Lepers R. Men cross America faster than women–the Race across America from 1982 to 2012. Int J Sports Physiol Perform. 2013;8(6):611–7. 10.1123/ijspp.8.6.611. Epub 2013/02/26. 23434612 10.1123/ijspp.8.6.611

[32] Rüst CA, Knechtle B, Knechtle P, Rosemann T. No case of exercise-associated hyponatraemia in top male ultra-endurance cyclists: the ‘Swiss Cycling Marathon’. Eur J Appl Physiol. 2012;112(2):689– 97. Epub 2011/06/10. 10.1007/s00421-011-2024-y. PubMed PMID: 21656229. 10.1007/s00421-011-2024-y 21656229

[33] Rüst CA, Bragazzi NL, Signori A, Stiefel M, Rosemann TJ, Knechtle B. Nation related participation and performance trends in ‘Norseman Xtreme Triathlon’ from 2006 to 2014. Springerplus. 2015;4. 10.1186/s40064-015-1255-5 PMC4556721 26357600

[34] Nikolaidis PT, Chtourou H, Ramirez-Campillo R, Villiger E, Rosemann T, Knechtle B. The Combined Effect of Aging and Performance Level on Pacing in Duathlon - the ITU Powerman Long Distance Duathlon World championships. Front Psychol. 2019;10:296. 10.3389/fpsyg.2019.00296. Epub 2019/03/06. 30833921 10.3389/fpsyg.2019.00296 PMC6388661

[35] Luk H-Y, McKenzie AL, Duplanty AA, Budnar RG, Levitt DE, Fernandez A, et al. Leukocyte subset changes in response to a 164-km road cycle ride in a hot environment. Int J Exerc Sci. 2016;9:34–46. 27293505 10.70252/FXYL1232 PMC4882474

[36] Lahart I, Lane A, Hulton A, Williams K, Godfrey RJ, Pedlar C, et al. Challenges in maintaining emotion regulation in a sleep and energy deprived State Induced by the 4800Km Ultra-endurance Bicycle race; the race across AMerica (RAAM). J Sports Sci Med. 2013;12:481–8. 24149155 PMC3772592

[37] Knechtle B, Wirth A, Knechtle P, Rüst CA, Rosemann TJ. A comparison of Ultra-endurance cyclists in a qualifying Ultra-cycling Race for Paris-Brest-Paris and Race across America—Swiss Cycling Marathon. Percept Mot Skills. 2012;114:110–96. 10.2466/05.PMS.114.1.96-110 22582679

[38] Knechtle B, Nikolaidis PT. Sex differences in pacing during ‘Ultraman Hawaii’. PeerJ. 2016;4:e2509. 10.7717. Epub 2016/10/06. 27703854 10.7717/peerj.2509 PMC5045888

[39] Knechtle B, Knechtle P, Andonie JL, Kohler G. Influence of anthropometry on race performance in extreme endurance triathletes: World Challenge Deca Iron Triathlon 2006. Br J Sports Med. 2007;41(10):644–8. 10.1136/bjsm.2006.035014. discussion 8. Epub 2007/06/09. 17556527 10.1136/bjsm.2006.035014 PMC2465179

[40] Chlíbková D, Rosemann TJ, Knechtle B, Nikolaidis PT, Žákovská A, Sudi K. Description of three female 24-h Ultra-endurance Race winners in various Weather conditions and disciplines. Chin J Physiol. 2017;60(4):231–41. 28847143 10.4077/CJP.2017.BAF443

[41] Gallmann D, Knechtle B, Rüst CA, Rosemann T, Lepers R. Elite triathletes in ‘Ironman Hawaii’ get older but faster. Age (Dordr). 2014;36(1):407–16. 10.1007/s11357-013-9534-y. Epub 2013/04/18. 23591938 10.1007/s11357-013-9534-y PMC3889914

[42] Knechtle B, Knechtle P, Lepers R. Participation and performance trends in ultra-triathlons from 1985 to 2009. Scand J Med Sci Sports. 2011;21(6):e82-90. Epub 2010/07/16. 10.1111/j.1600-0838.2010.01160.x. PubMed PMID: 20626703. 10.1111/j.1600-0838.2010.01160.x 20626703

[43] Knechtle B. Relationship of anthropometric and training characteristics with race performance in endurance and ultra-endurance athletes. Asian J Sports Med. 2014;5(2):73–90. Epub 2015/04/04. PubMed PMID: 25834701; PubMed Central PMCID: PMCPMC4374609. 25834701 PMC4374609

[44] Knechtle B, Knechtle P, Rosemann T. Similarity of anthropometric measures for male ultra-triathletes and ultra-runners. Percept Mot Skills. 2010;111(3):805–18. 10.2466/05.25.Pms.111.6.805-818. Epub 2011/02/16. 21319620 10.2466/05.25.PMS.111.6.805-818

[45] Knechtle B, Knechtle P, Rüst C, Rosemann T. A comparison of anthropometric and training characteristics of Ironman triathletes and Triple Iron ultra-triathletes. J Sports Sci. 2011;29:1373–80. 10.1080/02640414.2011.587442. 21834654 10.1080/02640414.2011.587442

[46] Knechtle B, Wirth A, Knechtle P, Rüst CA, Rosemann T. A comparison of ultra-endurance cyclists in a qualifying ultra-cycling race for Paris-Brest-Paris and Race Across America-Swiss cycling marathon. Percept Mot Skills. 2012;114(1):96–110. 10.2466/05.Pms.114.1.96-110. Epub 2012/05/16. 22582679 10.2466/05.PMS.114.1.96-110

[47] Knechtle B, Zingg MA, Rosemann T, Rüst CA. The aspect of experience in ultra-triathlon races. Springerplus. 2015;4(1):278. 10.1186/s40064-015-1050-3. 26101730 10.1186/s40064-015-1050-3 PMC4471069

[48] Stiefel M, Knechtle B, Lepers R. Master triathletes have not reached limits in their Ironman triathlon performance. Scand J Med Sci Sports. 2014;24. 10.1111/j.1600-0838.2012.01473.x 22582950

[49] Neumayr G, Gänzer H, Sturm W, Pfister R, Mitterbauer G, Hörtnagl H. Physiological effects of an Ultra-cycle ride in an amateur Athlete - A Case Report. J Sports Sci Med. 2002;1:20–6. 24672268 PMC3957577

[50] Rüst CA, Knechtle B, Knechtle P, Lepers R, Rosemann TJ, Onywera V. European athletes dominate performances in double Iron ultra-triathlons– A retrospective data analysis from 1985 to 2010. Eur J Sport Sci. 2014;14:S39 - S50. 10.1080/17461391.2011.641033 24111900

[51] Rüst CA, Knechtle B, Knechtle P, Rosemann TJ, Lepers R. Participation and performance trends in Triple Iron Ultra-triathlon– a cross-sectional and longitudinal data analysis. Asian J Sports Med. 2012;3:145–52. 23012633 10.5812/asjsm.34605 PMC3445641

[52] Sousa CV, Knechtle B, Nikolaidis PT. Longitudinal Performance Analysis in Ultra-Triathlon of the World’s 2 Best Master Triathletes. Int J Sports Physiol Perform. 2020:1–5. 10.1123/ijspp.2019-0805 32707562

[53] Sousa CV, Nikolaidis PT, Knechtle B. Ultra-triathlon—Pacing, performance trends, the role of nationality, and sex differences in finishers and non-finishers. Scand J Med Sci Sports. 2020;30(3):556–63. 10.1111/sms.13598. 31715049 10.1111/sms.13598

[54] Mavromati M, Kriemadis A, Gkatsis G, Psiloutsikou M, Koronios K. Motivation and high performance sports events: an exploratory investigation of the motives underlying repeated participation. Int J Sport Manage Mark. 2019;19:35. 10.1504/IJSMM.2019.10018016.

[55] Kruger M, Saayman M. How do mountain bikers and road cyclists differ? South African Journal for Research in Sport. Phys Educ Recreation. 2014;36:137–52.

[56] Kupchak BR, Kazman JB, Vingren JL, Levitt DE, Lee EC, Williamson KH, et al. Blood hemostatic changes during an Ultraendurance Road Cycling Event in a hot environment. Wilderness Environ Med. 2017;28(3):197–206. 10.1016/j.wem.2017.05.002. Epub 2017/07/26. 28739377 10.1016/j.wem.2017.05.002

[57] Knechtle B, Forte P, Weiss K, Cuk I, Nikolaidis PT, Sousa CV, et al. Biophysical characterization of the first ultra-cyclist in the world to break the 1,000 km barrier in 24-h non-stop road cycling: a case report. Front Cardiovasc Med. 2022;9:990382. 10.3389/fcvm.2022.990382. Epub 2022/10/29. 36304551 10.3389/fcvm.2022.990382 PMC9592711

[58] Allen SV, Hopkins WG. Age of peak competitive performance of Elite athletes: a systematic review. Sports Med. 2015;45(10):1431–41. 10.1007/s40279-015-0354-3. 26088954 10.1007/s40279-015-0354-3

[59] Knechtle B, Käch I, Rosemann T, Nikolaidis PT. The effect of sex, age and performance level on pacing of Ironman triathletes. Res Sports Med. 2019;27(1):99–111. PubMed PMID: 30418036. 30418036 10.1080/15438627.2018.1546703

[60] Lepers R, Rüst C, Stapley P, Knechtle B. Relative improvements in endurance performance with age: evidence from 25 years of Hawaii Ironman racing. Age (Dordrecht Netherlands). 2012;35. 10.1007/s11357-012-9392-z. 10.1007/s11357-012-9392-z PMC3636391 22367579

[61] Nikolaidis P, Villiger E, Ardigò L, Waśkiewicz Z, Rosemann T, Knechtle B. The age of peak performance in women and men duathletes - the paradigm of short and long versions in ‘Powerman Zofingen’. Open Access J Sports Med. 2018;9. 10.2147/OAJSM.S167735. 10.2147/OAJSM.S167735 PMC6054758 30140162

[62] Pozzi L, Knechtle B, Knechtle P, Rosemann T, Lepers R, Rüst CA. Sex and age-related differences in performance in a 24-hour ultra-cycling draft-legal event– a cross-sectional data analysis. BMC Sports Sci Med Rehabilitation. 2014;6(1):19. 10.1186/2052-1847-6-19. 10.1186/2052-1847-6-19 PMC4039327 24883191

[63] Frandsen J, Sahl RE, Rømer T, Hansen MT, Nielsen AB, Lie-Olesen MM, et al. Extreme duration exercise affects old and younger men differently. Acta Physiol (Oxf). 2022;235(3):e13816. 10.1111/apha.13816. Epub 2022/03/30. 35347845 10.1111/apha.13816 PMC9287057

[64] Gloor RU, Knechtle B, Knechtle P, Rüst CA, Haupt S, Rosemann T, et al. Sex-related trends in participation and performance in the ‘Swiss Bike Masters’ from 1994–2012. Percept Mot Skills. 2013;116(2):640–54. 10.2466/30.PMS.116.2.640-654. 24032336 10.2466/30.PMS.116.2.640-654

[65] Knechtle B, Zingg MA, Rosemann TJ, Rüst CA. Sex difference in top performers from Ironman to double deca iron ultra-triathlon. Open Access J Sports Med. 2014;5:159–72. 25114605 10.2147/OAJSM.S65977 PMC4079634

[66] Nikolaidis PT, Villiger E, Vancini RL, Rosemann T, Knechtle B. The Effect of Sex and Performance Level on Pacing in Duathlon. Sports (Basel). 2018;6(4). Epub 2018/11/28. 10.3390/sports6040152. PubMed PMID: 30477088; PubMed Central PMCID: PMCPMC6315520. 10.3390/sports6040152 PMC6315520 30477088

[67] Rüst CA, Knechtle B, Knechtle P, Rosemann T, Lepers R. Sex differences in Ultra-triathlon performance at increasing Race Distance. Percept Mot Skills. 2013;116(2):690–706. 10.2466/30.06.PMS.116.2.690-706. 24032340 10.2466/30.06.PMS.116.2.690-706

[68] Rüst CA, Rosemann T, Knechtle B. Performance and sex difference in ultra-triathlon performance from Ironman to double Deca Iron ultra-triathlon between 1978 and 2013. Springerplus [Internet] 2014. 2014;3:219. 10.1186/2193-1801-3-219 PMC4035499 24877030

[69] Rüst CA, Rosemann T, Lepers R, Knechtle B. Gender difference in cycling speed and age of winning performers in ultra-cycling - the 508-mile furnace Creek from 1983 to 2012. J Sports Sci. 2015;33(2):198–210. PubMed PMID: 24993112. 24993112 10.1080/02640414.2014.934705

[70] Salihu L, Rüst CA, Rosemann T, Knechtle B. Sex Difference in Draft-Legal Ultra-Distance Events - A Comparison between Ultra-Swimming and Ultra-Cycling. Chin J Physiol. 2016;59(2):87–99. Epub 2016/04/16. 10.4077/cjp.2016.Bae373. PubMed PMID: 27080464. 10.4077/CJP.2016.BAE373 27080464

[71] Baumgartner S, Sousa CV, Nikolaidis PT, Knechtle B. Can the Performance Gap between Women and Men be Reduced in Ultra-Cycling? Int J Environ Res Public Health. 2020;17(7). Epub 2020/04/11. 10.3390/ijerph17072521. PubMed PMID: 32272640; PubMed Central PMCID: PMCPMC7177769. 10.3390/ijerph17072521 PMC7177769 32272640

[72] Scholz H, Sousa CV, Baumgartner S, Rosemann T, Knechtle B. Changes in sex difference in Time-Limited Ultra-cycling races from 6 hours to 24 hours. Medicina. 2021;57(9):923. 10.3390/medicina57090923. PubMed PMID. 34577846 10.3390/medicina57090923 PMC8469116

[73] Barrero A, Erola P, Bescós R. Energy balance of triathletes during an ultra-endurance event. Nutrients. 2014;7(1):209–22. Epub 2015/01/07. doi: 10.3390/nu7010209. PubMed PMID: 25558906; PubMed Central PMCID: PMCPMC4303834. 25558906 10.3390/nu7010209 PMC4303834

[74] Bescós R, Rodríguez FA, Iglesias X, Benítez A, Marina M, Padullés JM, et al. High energy deficit in an ultraendurance athlete in a 24-hour ultracycling race. Proc (Bayl Univ Med Cent). 2012;25(2):124–8. PubMed PMID: 22481841; PubMed Central PMCID: PMCPMC3310508. 22481841 10.1080/08998280.2012.11928806 PMC3310508

[75] Bescós R, Rodríguez FA, Iglesias X, Knechtle B, Benítez A, Marina M, et al. Nutritional behavior of cyclists during a 24-hour team relay race: a field study report. J Int Soc Sports Nutr. 2012;9(1):3. 10.1186/1550-2783-9-3. 22309475 10.1186/1550-2783-9-3 PMC3287968

[76] Borgenvik M, Nordin M, Mikael Mattsson C, Enqvist JK, Blomstrand E, Ekblom B. Alterations in amino acid concentrations in the plasma and muscle in human subjects during 24 h of simulated adventure racing. Eur J Appl Physiol. 2012;112(10):3679–88. 10.1007/s00421-012-2350-8. Epub 2012/02/22. 22350359 10.1007/s00421-012-2350-8

[77] Chlíbková D, Knechtle B, Rosemann T, Tomášková I, Chadim V, Shortall M. Nutrition habits in 24-hour mountain bike racers. Springerplus. 2014;3:715. 10.1186/2193-1801-3-715. Epub 2015/02/13. 25674455 10.1186/2193-1801-3-715 PMC4320206

[78] Enqvist JK, Mattsson CM, Johansson PH, Brink-Elfegoun T, Bakkman L, Ekblom B. Energy turnover during 24 hours and 6 days of adventure racing. J Sports Sci. 2010;28:947–55. 20544486 10.1080/02640411003734069

[79] Francescato MP, di Prampero PE. Energy expenditure during an ultra-endurance cycling race. J Sports Med Phys Fit. 2002;42 1:1–7. 11832867

[80] Goedecke JH, Clark VR, Noakes TD, Lambert EV. The effects of medium-chain triacylglycerol and carbohydrate ingestion on ultra-endurance exercise performance. Int J Sport Nutr Exerc Metab. 2005;15(1):15–27. 10.1123/ijsnem.15.1.15. PubMed PMID: 15902986. 15902986 10.1123/ijsnem.15.1.15

[81] Konopka AR, Castor WM, Wolff CA, Musci RV, Reid JJ, Laurin JL, et al. Skeletal muscle mitochondrial protein synthesis and respiration in response to the energetic stress of an ultra-endurance race. J Appl Physiol. 2017;123 6:1516–24. 28883046 10.1152/japplphysiol.00457.2017 PMC6157642

[82] Koopman R, Pannemans D, Jeukendrup AE, Gijsen AP, Senden JM, Halliday D, et al. Combined ingestion of protein and carbohydrate improves protein balance during ultra-endurance exercise. Am J Physiol Endocrinol Metabolism. 2004;287(4):E712–20. 10.1152/ajpendo.00543.2003 15165999

[83] Maunder E, Kilding AE, Plews DJ. Substrate metabolism during Ironman Triathlon: different horses on the same courses. Sports Med. 2018;48(10):2219–26. 10.1007/s40279-018-0938-9. Epub 2018/05/20. 29777386 10.1007/s40279-018-0938-9

[84] Nikolaidis PT, Veniamakis E, Rosemann T, Knechtle B. Nutrition in Ultra-endurance: state of the art. Nutrients. 2018;10(12):1995. 10.3390/nu10121995. PubMed PMID:. 30558350 10.3390/nu10121995 PMC6315825

[85] Rowlands DS, Hopkins WG. Effects of high-fat and high-carbohydrate diets on metabolism and performance in cycling. Metabolism. 2002;51(6):678–90. 10.1053/meta.2002.32723. Epub 2002/05/31. 12037719 10.1053/meta.2002.32723

[86] Wilson PB, Rhodes GS, Ingraham SJ. Saccharide Composition of Carbohydrates Consumed during an Ultra-endurance Triathlon. J Am Coll Nutr. 2015;34:497–506. 25941980 10.1080/07315724.2014.996830

[87] Knechtle B, Pitre J, Chandler C. Food habits and use of supplements in ultraendurance cyclists - the race across AMerica (RAAM) 2006. Sportmed Sporttraumatologie. 2007;55.

[88] Armstrong LE, Casa DJ, Emmanuel H, Ganio MS, Klau JF, Lee EC, et al. Nutritional, physiological, and perceptual responses during a summer ultraendurance cycling event. J Strength Cond Res. 2012;26(2):307–18. 10.1519/JSC.0b013e318240f677. Epub 2011/11/23. 22105054 10.1519/JSC.0b013e318240f677

[89] Black KE, Skidmore PM, Brown RC. Energy intakes of ultraendurance cyclists during competition, an observational study. Int J Sport Nutr Exerc Metab. 2012;22(1):19–23. 10.1123/ijsnem.22.1.19. Epub 2012/01/18. 22248496 10.1123/ijsnem.22.1.19

[90] Hulton AT, Lahart I, Williams KL, Godfrey R, Charlesworth S, Wilson M, et al. Energy expenditure in the Race across America (RAAM). Int J Sports Med. 2010;31(7):463–7. 10.1055/s-0030-1251992. Epub 2010/05/11. 20455193 10.1055/s-0030-1251992

[91] Armstrong LE, Johnson EC, Ganio MS, Judelson DA, Vingren JL, Kupchak BR, et al. Effective body water and body mass changes during summer ultra-endurance road cycling. J Sports Sci. 2015;33:125–35. 24992367 10.1080/02640414.2014.932918

[92] Gauckler P, Kesenheimer JS, Kronbichler A, Kolbinger FR. Edema-like symptoms are common in ultra-distance cyclists and driven by overdrinking, use of analgesics and female sex - a study of 919 athletes. J Int Soc Sports Nutr. 2021;18(1):73. 10.1186/s12970-021-00470-0. Epub 2021/12/06. 34863204 10.1186/s12970-021-00470-0 PMC8643017

[93] Goulet EDB, Hoffman MD. Impact of Ad Libitum Versus Programmed Drinking on Endurance Performance: A Systematic Review with Meta-Analysis. Sports Med. 2019;49(2):221– 32. Epub 2019/01/20. 10.1007/s40279-018-01051-z. PubMed PMID: 30659500. 10.1007/s40279-018-01051-z 30659500

[94] Knechtle B, Chlíbková D, Nikolaidis PT. [Exercise-Associated hyponatremia in endurance performance]. Praxis. 2019;108(9):615–32. 10.1024/1661-8157/a003261. PubMed PMID: 31288661. 31288661 10.1024/1661-8157/a003261

[95] Knechtle B, Chlíbková D, Papadopoulou S, Mantzorou M, Rosemann T, Nikolaidis PT. Exercise-Associated Hyponatremia in endurance and Ultra-endurance performance-aspects of sex, race location, ambient temperature, sports Discipline, and length of performance: a narrative review. Medicina (Kaunas) [Internet]. 2019 2019/08//; 55(9):[E537 p.]. 10.3390/medicina55090537 PMC6780610 31455034

[96] Meyer M, Knechtle B, Bürge J, Knechtle P, Mrazek C, Wirth A, et al. Ad libitum fluid intake leads to no leg swelling in male Ironman triathletes - an observational field study. J Int Soc Sports Nutr. 2012;9(1):40. 10.1186/1550-2783-9-40. Epub 2012/09/04. 22937792 10.1186/1550-2783-9-40 PMC3524467

[97] Moyen NE, Ganio MS, Wiersma LD, Kavouras SA, Gray M, McDermott BP, et al. Hydration status affects mood state and pain sensation during ultra-endurance cycling. J Sports Sci. 2015;33:1962–9. 25793570 10.1080/02640414.2015.1021275

[98] Rüst CA, Knechtle B, Knechtle P, Rosemann TJ. Higher prevalence of exercise-associated hyponatremia in triple iron ultra-triathletes than reported for ironman triathletes. Chin J Physiol. 2012;55(3):147–55. 22784278 10.4077/CJP.2012.BAA010

[99] Knechtle B, Gnädinger M, Knechtle P, Imoberdorf R, Kohler G, Ballmer P, et al. Prevalence of exercise-associated hyponatremia in male ultraendurance athletes. Clin J Sport Med. 2011;21(3):226–32. 10.1097/JSM.0b013e31820cb021. Epub 2011/03/24. 21427567 10.1097/JSM.0b013e31820cb021

[100] Pollastri L, Lanfranconi F, Tredici G, Burtscher M, Gatterer H. Body Water Status and short-term maximal power output during a Multistage Road Bicycle race (Giro d’Italia 2014). Int J Sports Med. 2016;37(4):329–33. 10.1055/s-0035-1565105. Epub 2015/12/25. 26701829 10.1055/s-0035-1565105

[101] Black KE, Skidmore P, Brown RC. Fluid balance of cyclists during a 387-km race. Eur J Sport Sci. 2014;14(Suppl 1):S421–8. PubMed PMID: 24444237. Epub 2014/01/22. 24444237 10.1080/17461391.2012.711860

[102] Bischof M, Knechtle B, A.Rüst C, Knechtle P, Rosemann TJ. Changes in Skinfold thicknesses and Body Fat in Ultra-endurance cyclists. Asian J Sports Med. 2013;4:15–22. 23785571 PMC3685155

[103] Knechtle B, Andonie JL, Salas OF, Knechtle P, Kohler G. [The effect of a multi-stage ultra-endurance triathlon over ten times an iron-man-triathlon on fat mass and skeletal muscle mass–the world challenge deca iron 2006]. Praxis. 2008;97:16885–92. 10.1024/1661-8157.97.16.885 18777716

[104] Knechtle B, Knechtle P, Schück R, Andonie JL, Kohler G. Effects of a Deca Iron Triathlon on body composition: a case study. Int J Sports Med. 2008;29(4):343–51. 10.1055/s-2007-965354. Epub 2007/09/21. 17879892 10.1055/s-2007-965354

[105] Knechtle B, Schwanke M, Knechtle P, Kohler G. Decrease in body fat during an ultra-endurance triathlon is associated with race intensity. Br J Sports Med. 2008;42(7):609–13. 10.1136/bjsm.2007.040956. Epub 2007/12/01. 18048432 10.1136/bjsm.2007.040956

[106] Hue O, Voltaire B, Hertogh C, Blonc S. Heart rate, thermoregulatory and humoral responses during a 9-day cycle race in a hot and humid climate. Int J Sports Med. 2006;27(9):690–6. 10.1055/s-2005-872919. Epub 2006/04/06. 16586332 10.1055/s-2005-872919

[107] White JA, Ward C, Nelson H. Ergogenic demands of a 24 hour cycling event. Br J Sports Med. 1984;18(3):165–71. 10.1136/bjsm.18.3.165. Epub 1984/09/01. 6487942 10.1136/bjsm.18.3.165 PMC1859384

[108] Barrero A, Chaverri D, Erola P, Iglesias X, Rodríguez FA. Intensity profile during an ultra-endurance triathlon in relation to testing and performance. Int J Sports Med. 2014;35(14):1170–8. 10.1055/s-0034-1374601. Epub 2014/09/12. 25210791 10.1055/s-0034-1374601

[109] Berglund B, Berglund L. [»Shermer’s neck« is a rare injury in long-distance cycle races. Association with diplopia described for the first time]. Lakartidningen [Internet]. 2015 2015/12//; 112:[DR7I p.]. http://europepmc.org/abstract/MED/26671432. 26671432

[110] Brayson D, Frigiola A, Clark JE. Dynamic heart rate response to multi-day unsupported ultra‐endurance cycle racing: a case report. Exp Physiol. 2019;104:174–9. 30582664 10.1113/EP087341

[111] Chlíbková D, Ronzhina M, Nikolaidis PT, Rosemann T, Knechtle B. Non-steroidal anti-inflammatory drug consumption in a Multi-stage and a 24-h Mountain Bike Competition. Front Physiol. 2018. 10.3389/fphys.2018.01272. 9:1272. Epub 2018/09/25. 30246809 10.3389/fphys.2018.01272 PMC6139357

[112] Devrim-Lanpir A, Bilgic P, Kocahan T, Deliceoğlu G, Rosemann T, Knechtle B. Total dietary antioxidant intake including Polyphenol Content: is it capable to fight against increased oxidants within the body of Ultra-endurance athletes? Nutrients [Internet]. 2020 2020/06//; 12(6):[E1877 p.]. 10.3390/nu12061877 PMC7353279 32586010

[113] Gamage PJ, Fortington LV, Finch C. Epidemiology of exertional heat illnesses in organised sports: a systematic review. J Sci Med Sport. 2019. 10.1016/j.jsams.2020.02.008 32144023

[114] Knechtle B, Kohler G. Running performance, not anthropometric factors, is associated with race success in a Triple Iron Triathlon. Br J Sports Med. 2009;43(6):437–41. 10.1136/bjsm.2007.039602. Epub 2007/12/01. 18048440 10.1136/bjsm.2007.039602

[115] Knechtle B, Zingg MA, Rosemann TJ, Stiefel M, Rüst CA. What predicts performance in ultra-triathlon races?– a comparison between Ironman distance triathlon and ultra-triathlon. Open Access J Sports Med. 2015;6:149–59. 26056498 10.2147/OAJSM.S79273 PMC4445872

[116] Knechtle B, Bragazzi NL, Rosemann T, Rüst CA. Pacing in a self-paced world record attempt in 24-h road cycling. Springerplus [Internet]. 2015 2015; 4:[650 p.]. 10.1186/s40064-015-1445-1 PMC4628007 26543784

[117] Knechtle B, Wirth A, Knechtle P, Rüst CA, Rosemann T, Lepers R. No improvement in race performance by naps in male ultra-endurance cyclists in a 600-km ultra-cycling race. Chin J Physiol. 2012;55(2):125–33. 10.4077/cjp.2012.Baa022. Epub 2012/05/09. PubMed PMID: 22559737. 22559737 10.4077/CJP.2012.BAA022

[118] Lucas SJ, Anglem N, Roberts WS, Anson JG, Palmer CD, Walker RJ, et al. Intensity and physiological strain of competitive ultra-endurance exercise in humans. J Sports Sci. 2008;26(5):477–89. Epub 2008/02/16. doi: 10.1080/02640410701552872. PubMed PMID: 18274945. 18274945 10.1080/02640410701552872

[119] Luk H-Y, Levitt DE, Lee EC-H, Ganio MS, McDermott BP, Kupchak BR, et al. Pro- and anti-inflammatory cytokine responses to a 164-km road cycle ride in a hot environment. Eur J Appl Physiol. 2016;116:2007–15. 27522585 10.1007/s00421-016-3452-5

[120] Vingren JL, Budnar RG, McKenzie AL, Duplanty AA, Luk H-Y, Levitt DE, et al. The acute testosterone, growth hormone, cortisol and interleukin-6 response to 164-km road cycling in a hot environment. J Sports Sci. 2016;34:694–9. 26199143 10.1080/02640414.2015.1068440

[121] Mattsson CM, Enqvist JK, Brink-Elfegoun T, Johansson PH, Bakkman L, Ekblom B. Reversed drift in heart rate but increased oxygen uptake at fixed work rate during 24 h ultra-endurance exercise. Scand J Med Sci Sports. 2010;20(2):298–304. Epub 2009/06/03. 10.1111/j.1600-0838.2009.00878.x. PubMed PMID: 19486489. 10.1111/j.1600-0838.2009.00878.x 19486489

[122] Millet GY, Martin V, Temesi J. The role of the nervous system in neuromuscular fatigue induced by ultra-endurance exercise. Appl Physiol Nutr Metab. 2018;43(11):1151–7. 10.1139/apnm-2018-0161. PubMed PMID: 29726694. 29726694 10.1139/apnm-2018-0161

[123] Rogers IR, Speedy D, Hillman D, Noffsinger B, Inglis S, Wilderness. Environ Med. 2002;13(2):135–9. 10.1580/1080-6032(2002)013[0135:RFCIAW]2.0.CO;2. 10.1580/1080-6032(2002)013[0135:rfciaw]2.0.co;2 12092967

[124] Smith KA, Kisiolek JN, Willingham BD, Morrissey MC, Leyh SM, Saracino PG, et al. Ultra-endurance triathlon performance and markers of whole-body and gut-specific inflammation. Eur J Appl Physiol. 2019;120:349–57. 31828478 10.1007/s00421-019-04279-3

[125] Sousa CV, Pereira RW, Rosemann T, Nikolaidis PT, Knechtle B. Self-selected pacing during a World Record Attempt in 40 Ironman-Distance triathlons in 40 days. Int J Environ Res Public Health [Internet]. 2020 2020/04//; 17(7):E2390 p.]. 10.3390/ijerph17072390 PMC7177248 32244582

[126] Knechtle B, Knechtle P, Rosemann T. No Association between Skinfold thicknesses and Race Performance in Male Ultra-endurance cyclists in a 600 km Ultra-cycling Marathon. Hum Mov. 2009;10(2):91–5. 10.2478/v10038-009-0018-y.

[127] Knechtle B, Knechtle P, Rüst CA, Rosemann T, Lepers R. Finishers and nonfinishers in the ‘Swiss Cycling Marathon' to qualify for the ‘Race across America ‘. J Strength Cond Res. 2011;25(12):3257–63. 10.1519/JSC.0b013e31821606b3. Epub 2011/11/15. 22080313 10.1519/JSC.0b013e31821606b3

[128] Bell PG, Furber MJ, Antón-Solanas KAVANS, Swart A. The physiological Profile of a multiple Tour De France winning cyclist. Med Sci Sports Exerc. 2017;49(1):115–23. 10.1249/mss.0000000000001068. Epub 2016/08/11. 27508883 10.1249/MSS.0000000000001068

[129] Almar M, Villa JG, Cuevas MJ, Rodríguez-Marroyo JA, Avila C, Gonzalez-Gallego J. Urinary levels of 8-hydroxydeoxyguanosine as a marker of oxidative damage in road cycling. Free Radic Res. 2002;36(3):247–53. Epub 2002/06/20. doi: 10.1080/10715760290019255. PubMed PMID: 12071342. 12071342 10.1080/10715760290019255

[130] Kupchak BR, McKenzie AL, Luk HY, Saenz C, Kunces LJ, Ellis LA et al. Effect of cycling in the heat for 164 km on procoagulant and fibrinolytic parameters. Eur J Appl Physiol. 2015;115(6):1295– 303. Epub 2015/01/22. 10.1007/s00421-015-3107-y. PubMed PMID: 25603777. 10.1007/s00421-015-3107-y 25603777

[131] Colombini A, Corsetti R, Machado M, Graziani R, Lombardi G, Lanteri P et al. Serum creatine kinase activity and its relationship with renal function indices in professional cyclists during the Giro d’Italia 3-week stage race. Clin J Sport Med. 2012;22(5):408– 13. Epub 2012/06/30. 10.1097/JSM.0b013e31825e66cc. PubMed PMID: 22744001. 10.1097/JSM.0b013e31825e66cc 22744001

[132] Lombardi G, Lanteri P, Fiorella PL, Simonetto L, Impellizzeri FM, Bonifazi M, et al. Comparison of the hematological profile of elite road cyclists during the 2010 and 2012 GiroBio ten-day stage races and relationships with final ranking. PLoS ONE. 2013;8(4):e63092. 10.1371/journal.pone.0063092. Epub 2013/05/07. 23646180 10.1371/journal.pone.0063092 PMC3639959

[133] Menaspà P, Abbiss CR, Martin DT. Performance analysis of a world-class sprinter during cycling grand tours. Int J Sports Physiol Perform. 2013;8(3):336–40. 10.1123/ijspp.8.3.336. Epub 2012/10/06. 23038704 10.1123/ijspp.8.3.336

[134] Neumayr G, Pfister R, Hoertnagl H, Mitterbauer G, Prokop W, Joannidis M. Renal function and plasma volume following ultramarathon cycling. Int J Sports Med. 2005;26(1):2–8. 10.1055/s-2004-815717. Epub 2005/01/12. 15643528 10.1055/s-2004-815717

[135] Chlíbková D, Knechtle B, Rosemann T, Tomášková I, Novotný J, Žákovská A, et al. Rhabdomyolysis and exercise-associated hyponatremia in ultra-bikers and ultra-runners. J Int Soc Sports Nutr. 2015. 10.1186/s12970-015-0091-x. 12:29. Epub 2015/06/27. 26113805 10.1186/s12970-015-0091-x PMC4480906

[136] Nédélec M, Chauvineau M, Guilhem G. On the Road to Camarón: The Sleep of an Ultra-Endurance Athlete Cycling 10,000 km in 24 Days. Int J Environ Res Public Health. 2022;19(8). Epub 2022/04/24. 10.3390/ijerph19084543. PubMed PMID: 35457410; PubMed Central PMCID: PMCPMC9025025. 10.3390/ijerph19084543 PMC9025025 35457410

[137] Irvine D, Jobson SA, Wilson JP. Evaluating Changes in Mental Workload in Indoor and Outdoor Ultra-Distance Cycling. Sports (Basel). 2022;10(5). Epub 2022/05/28. 10.3390/sports10050067. PubMed PMID: 35622476; PubMed Central PMCID: PMCPMC9146483. 10.3390/sports10050067 PMC9146483 35622476

[138] Manunzio C, Mester J, Kaiser W, Wahl P. Training intensity distribution and changes in performance and physiology of a 2nd place Finisher Team of the race across America over a 6 Month Preparation Period. Front Physiol. 2016;7:642. 10.3389/fphys.2016.00642. Epub 2017/01/14. 28082909 10.3389/fphys.2016.00642 PMC5187238

[139] Rothschild JA, Delcourt M, Maunder E, Plews DJ. Racing and training physiology of an Elite Ultra-endurance Cyclist: Case Study of 2 record-setting performances. Int J Sports Physiol Perform. 2021;16(5):739–43. 10.1123/ijspp.2020-0515. Epub 2021/02/07. 33547258 10.1123/ijspp.2020-0515

[140] Kraemer WJ, Fragala MS, Watson G, Volek JS, Rubin MR, French DN, et al. Hormonal responses to a 160-km race across frozen Alaska. Br J Sports Med. 2008;42(2):116–20. 10.1136/bjsm.2007.035535. discussion 20. Epub 2007/07/20. 17638844 10.1136/bjsm.2007.035535

[141] Geesmann B, Gibbs JC, Mester J, Koehler K. Association between Energy Balance and metabolic hormone suppression during Ultraendurance Exercise. Int J Sports Physiol Perform. 2017;12(7):984–9. 10.1123/ijspp.2016-0061. Epub 2016/12/15. 27967268 10.1123/ijspp.2016-0061

[142] El Helou N, Berthelot G, Thibault V, Tafflet M, Nassif H, Campion F, et al. Tour De France, Giro, Vuelta, and classic European races show a unique progression of road cycling speed in the last 20 years. J Sports Sci. 2010;28:789–96. 10.1080/02640411003739654. 20473822 10.1080/02640411003739654

[143] Knechtle B, Rosemann T, Nikolaidis PT. Self-Selected Pacing during a 24 h Track Cycling World Record. Int J Environ Res Public Health. 2019;16(16). Epub 2019/08/21. 10.3390/ijerph16162943. PubMed PMID: 31426293; PubMed Central PMCID: PMCPMC6720958. 10.3390/ijerph16162943 PMC6720958 31426293

[144] Garvican-Lewis LA, Clark B, Martin DT, Schumacher YO, McDonald W, Stephens B, et al. Impact of Altitude on Power output during Cycling Stage Racing. PLoS ONE. 2015;10(12):e0143028. 10.1371/journal.pone.0143028. Epub 2015/12/03. 26629912 10.1371/journal.pone.0143028 PMC4668098

[145] Fernández-García B, Pérez-Landaluce J, Rodríguez-Alonso M, Terrados N. Intensity of exercise during road race pro-cycling competition. Med Sci Sports Exerc. 2000;32(5):1002–6. 10.1097/00005768-200005000-00019. Epub 2000/05/05. 10795793 10.1097/00005768-200005000-00019

[146] Luciá A, Hoyos J, Carvajal A, Chicharro JL. Heart rate response to professional road cycling: the Tour De France. Int J Sports Med. 1999;20(3):167–72. 10.1055/s-1999-970284. Epub 1999/05/20. 10333093 10.1055/s-1999-970284

[147] Lucía A, Hoyos J, Pérez M, Santalla A, Chicharro JL. Inverse relationship between VO2max and economy/efficiency in world-class cyclists. Med Sci Sports Exerc. 2002;34(12):2079–84..Df. PubMed PMID: 12471319. 12471319 10.1249/01.MSS.0000039306.92778.DF

[148] Lucía A, Rabadán M, Hoyos J, Hernández-Capilla M, Pérez M, San Juan AF, et al. Frequency of the VO2max plateau phenomenon in world-class cyclists. Int J Sports Med. 2006;27(12):984–92. 10.1055/s-2006-923833. Epub 2006/06/02. 16739087 10.1055/s-2006-923833

[149] Rüst CA, Knechtle B, Rosemann TJ, Lepers R. Men cross America faster than women–the Race across America from 1982 to 2012. Int J Sports Physiol Perform. 2013;8 6:611–7. 23434612 10.1123/ijspp.8.6.611

[150] Kholkine L, Servotte T, de Leeuw AW, De Schepper T, Hellinckx P, Verdonck T, et al. A learn-to-Rank Approach for Predicting Road Cycling race outcomes. Front Sports Act Living. 2021;3:714107. 10.3389/fspor.2021.714107. Epub 2021/10/26. 34693282 10.3389/fspor.2021.714107 PMC8527032

[151] RCS_Mediagroup M-S. 2023 2024 [10.02.2024]. https://www.milanosanremo.it/en/live-archive/tappa/2023/1/.

[152] Peloton. Ronde van Flaanderen Cyclo 2024 [10.02.2024]. https://www.rondevanvlaanderen.be/en.

[153] Amstel. Amstel Gold Race 2024 [10.02.2024]. https://www.amstel.nl/amstelgoldrace.

[154] Shoak MA, Knechtle B, Knechtle P, Rüst CA, Rosemann T, Lepers R. Participation and performance trends in ultracycling. Open Access J Sports Med. 2013;4:41–51. 10.2147/oajsm.S40142. Epub 2014/01/01. 24379708 10.2147/OAJSM.S40142 PMC3871902

[155] Käch IW, Rüst CA, Nikolaidis PT, Rosemann T, Knechtle B. The age-related performance decline in Ironman Triathlon starts earlier in Swimming Than in Cycling and running. J Strength Cond Res. 2018;32(2):379–95. 10.1519/jsc.0000000000001796. Epub 2017/02/23. 28225523 10.1519/JSC.0000000000001796

[156] Pozzi L, Knechtle B, Knechtle P, Rosemann T, Lepers R, Rüst CA. Sex and age-related differences in performance in a 24-hour ultra-cycling draft-legal event - a cross-sectional data analysis. BMC Sports Sci Med Rehabil. 2014;6:19. 10.1186/2052-1847-6-19. Epub 2014/06/03. 24883191 10.1186/2052-1847-6-19 PMC4039327

[157] Carpenter J, Weinstein S, Myseros J, Vezina G, Bell MJ. Inadvertent hyponatremia leading to acute cerebral edema and early evidence of herniation. Neurocrit Care. 2007;6(3):195-9. Epub 2007/06/19. 10.1007/s12028-007-0032-x. PubMed PMID: 17572863. 10.1007/s12028-007-0032-x 17572863

[158] Verbrugge F, Steels P, Grieten L, Nijst P, Tang W, Mullens W. Hyponatremia in Acute Decompensated Heart failure depletion Versus Dilution. J Am Coll Cardiol. 2015;65:480–92. 10.1016/j.jacc.2014.12.010. 25660927 10.1016/j.jacc.2014.12.010

[159] Kheetan M, Ogu I, Shapiro JI, Khitan ZJ. Acute and Chronic Hyponatremia. Front Med. 2021;8. 10.3389/fmed.2021.693738. 10.3389/fmed.2021.693738 PMC8369240 34414205

[160] Rosner MH. Exercise-associated hyponatremia. Trans Am Clin Climatol Assoc. 2019;130:76–87. PubMed PMID: 31516170. 31516170 PMC6735969

[161] Chorley J, Cianca J, Divine J. Risk factors for exercise-associated hyponatremia in non-elite marathon runners. Clin J Sport Med. 2007;17(6):471–7. 10.1097/JSM.0b013e3181588790. Epub 2007/11/13. 17993790 10.1097/JSM.0b013e3181588790

[162] Noakes TD. Is drinking to thirst optimum? Ann Nutr Metab. 2010;57(Suppl 2):9–17. Epub 2010/01/01. doi: 10.1159/000322697. PubMed PMID: 21346332. 21346332 10.1159/000322697

[163] Noakes TD, Adams BA, Myburgh KH, Greeff C, Lotz T, Nathan M. The danger of an inadequate water intake during prolonged exercise. A novel concept re-visited. Eur J Appl Physiol Occup Physiol. 1988;57(2):210–9. 10.1007/bf00640665. Epub 1988/01/01. 3349989 10.1007/BF00640665

[164] Königstein K, Niess AM, Carlsohn A, Treff G. Hydration Management in sports. Dtsch Z für Sportmedizin. 2022;73(4):137–41.

[165] Dugas JP, Oosthuizen U, Tucker R, Noakes TD. Rates of fluid ingestion alter pacing but not thermoregulatory responses during prolonged exercise in hot and humid conditions with appropriate convective cooling. Eur J Appl Physiol. 2009;105(1):69–80. 10.1007/s00421-008-0876-6. Epub 2008/10/15. 18853180 10.1007/s00421-008-0876-6

[166] Backx K, van Someren KA, Palmer GS. One hour cycling performance is not affected by ingested fluid volume. Int J Sport Nutr Exerc Metab. 2003;13(3):333–42. 10.1123/ijsnem.13.3.333. Epub 2003/12/13. 14669933 10.1123/ijsnem.13.3.333

[167] Bardis CN, Kavouras SA, Adams JD, Geladas ND, Panagiotakos DB, Sidossis LS. Prescribed drinking leads to Better Cycling Performance than Ad Libitum Drinking. Med Sci Sports Exerc. 2017;49(6):1244–51. 10.1249/mss.0000000000001202. Epub 2017/01/13. 28079705 10.1249/MSS.0000000000001202

[168] Knechtle B, Enggist A, Jehle T. Energy turnover at the Race across AMerica (RAAM) - a case report. Int J Sports Med. 2005;26(6):499–503. 10.1055/s-2004-821136. Epub 2005/07/23. 16037895 10.1055/s-2004-821136

[169] Raschka C, Plath M. [Body fat compartment and its relationship to food intake and clinical chemical parameters during extreme endurance performance]. Schweiz Z Sportmed. 1992;40(1):13–25. Epub 1992/03/01. PubMed PMID: 1561538. 1561538

[170] Zehnder M, Ith M, Kreis R, Saris W, Boutellier U, Boesch C. Gender-specific usage of intramyocellular lipids and glycogen during exercise. Med Sci Sports Exerc. 2005;37(9):1517-24. Epub 2005/09/24. 10.1249/01.mss.0000177478.14500.7c. PubMed PMID: 16177603. 10.1249/01.mss.0000177478.14500.7c 16177603

[171] Khodaee M, Luyten D, Hew-Butler T. Exercise-Associated Hyponatremia in an Ultra-endurance Mountain Biker A Case Report. Sports Health: Multidisciplinary Approach. 2013;5:334–6. 10.1177/1941738113480928. 10.1177/1941738113480928 PMC3899906 24459549

[172] Del Campo A, Contreras-Hernández I, Castro-Sepúlveda M, Campos CA, Figueroa R, Tevy MF, et al. Muscle function decline and mitochondria changes in middle age precede Sarcopenia in mice. Aging. 2018;10(1):34–55. 10.18632/aging.101358. Epub 2018/01/06. 29302020 10.18632/aging.101358 PMC5811241

[173] Seo DY, Lee SR, Kim N, Ko KS, Rhee BD, Han J. Age-related changes in skeletal muscle mitochondria: the role of exercise. Integr Med Res. 2016;5(3):182–6. 10.1016/j.imr.2016.07.003. 28462116 10.1016/j.imr.2016.07.003 PMC5390452

[174] Noakes TD, Gibson ASC, Lambert EV. From catastrophe to complexity: a novel model of integrative central neural regulation of effort and fatigue during exercise in humans: summary and conclusions. Br J Sports Med. 2005;39(2):120–4. 10.1136/bjsm.2003.010330. 15665213 10.1136/bjsm.2003.010330 PMC1725112

